# Mechanical instability of monocrystalline and polycrystalline methane hydrates

**DOI:** 10.1038/ncomms9743

**Published:** 2015-11-02

**Authors:** Jianyang Wu, Fulong Ning, Thuat T. Trinh, Signe Kjelstrup, Thijs J. H. Vlugt, Jianying He, Bjørn H. Skallerud, Zhiliang Zhang

**Affiliations:** 1 Department of Physics, Research Institute for Biomimetics and Soft Matter, Xiamen University, Xiamen 361005, China; 2 Faculty of Engineering Science and Technology, Norwegian University of Science and Technology, 7491 Trondheim, Norway; 3 Faculty of Engineering, China University of Geosciences, Wuhan, Hubei 430074, China; 4 Faculty of Natural Sciences and Technology, Norwegian University of Science and Technology, 7491 Trondheim, Norway; 5 Process and Energy Laboratory, Delft University of Technology, Leeghwaterstraat 39, 2628CB Delft, The Netherlands

## Abstract

Despite observations of massive methane release and geohazards associated with gas hydrate instability in nature, as well as ductile flow accompanying hydrate dissociation in artificial polycrystalline methane hydrates in the laboratory, the destabilising mechanisms of gas hydrates under deformation and their grain-boundary structures have not yet been elucidated at the molecular level. Here we report direct molecular dynamics simulations of the material instability of monocrystalline and polycrystalline methane hydrates under mechanical loading. The results show dislocation-free brittle failure in monocrystalline hydrates and an unexpected crossover from strengthening to weakening in polycrystals. Upon uniaxial depressurisation, strain-induced hydrate dissociation accompanied by grain-boundary decohesion and sliding destabilises the polycrystals. In contrast, upon compression, appreciable solid-state structural transformation dominates the response. These findings provide molecular insight not only into the metastable structures of grain boundaries, but also into unusual ductile flow with hydrate dissociation as observed during macroscopic compression experiments.

## Abstract (web summary)

Sediment-hosted gas hydrates may release vast quantities of methane upon failure, but destabilizing mechanisms at the molecular level are poorly understood. Here, the authors study the deformation using simulations and find that failure differs between single crystals and polycrystalline hydrates.

## Graphical abstract

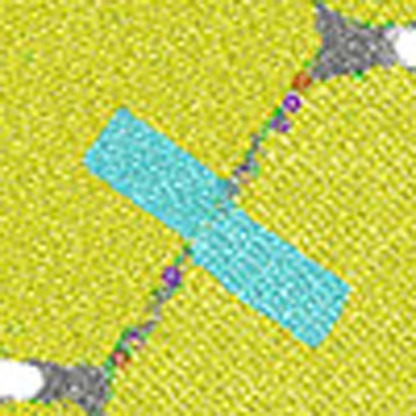

Natural gas hydrates are ice-like crystalline substances in which gas molecules are physically trapped inside the polyhedral cavities of water molecules^1^,^2^,^3^. There are three common types of gas hydrate structures: sI hydrate, sII hydrate and sH hydrate^1^. They occur abundantly in both petrochemical production lines^4^ and hostile environments such as seafloor sediments, arctic or permafrost regions and even the surfaces of other planets^5^,^6^. In a natural environment, the sI structure is the most common form of gas hydrates in which methane is the main hydrate former^1^. Therefore, natural gas hydrates have attracted great attention from academia, industry and the government due to their importance in flow assurance^1^,^4^, energy technology (including hydrogen storage)^7^,^8^,^9^ and environmental and climate issues^10^,^11^,^12^,^13^,^14^. Similarly to conventional solid materials (for example, ceramics, metals and low-dimensional graphene), gas hydrates in both natural and laboratory settings are grain-textured polycrystalline icy compounds^15^. The geomechanical stability of gas-hydrate-bearing sediments is strongly reflected by the stability of the gas hydrate phase within the formation^16^, which indicates that natural gas hydrates possess profound implications for seabed stability, the safety of petro-activities and future trapped gas recovery, and planetary evolution scenarios^17^,^18^. Driven by these issues of practical importance, numerous attempts have been made over the past decades to address research questions concerning hydrate stability conditions and the quantification of their physico-chemical properties^12^,^13^,^14^,^18^,^19^. Unfortunately, the low availability of high-quality gas hydrate samples recovered from nature and synthesized in the laboratory strongly limits the study of their properties. Therefore, the properties of water ice have been commonly assumed as a proxy for gas hydrates^17^.

However, many investigations have indicated that the thermal and mechanical properties of gas hydrates are different from those of ice^20^,^21^,^22^,^23^,^24^,^25^. For instance, depending on the temperature, the thermal conductivity of clathrate hydrates is 4- to 20-fold lower than that of water ice^25^. Extremely large anharmonic motions of the guest molecules lead to peculiar thermal transport behaviour in clathrate hydrate systems^26^. Mechanically, under uniaxial compression, laboratory-formed polycrystalline methane hydrates are 20- to 40-fold stronger than ice (I_*h*_)^20^,^21^ and experience extensive strain hardening followed by strain softening^21^,^23^. Local solid-state methane hydrate exsolution or dissociation was observed during a compressive process, even under pressure and temperature conditions well within the methane hydrate stability zone^20^,^22^,^23^. However, because the currently available direct experimental nano-visualization techniques for methane hydrates are insufficient, the intrinsic mechanism of the ductile flow behaviour of polycrystalline methane hydrates, accompanied by solid-state hydrate exsolution or dissociation induced by cold deformation, remains poorly understood at the molecular level. Despite great advancements in the understanding of the grain-size effect on the intrinsic mechanical properties of conventional polycrystalline materials at the nanoscale, the effect of grain size on the failure behaviour of polycrystalline methane hydrates has not yet been reported. In addition, molecular-level structures of the grain boundaries of polycrystalline methane hydrates have not yet been identified. So far, classic molecular dynamics (MD) simulation as an indispensable tool has been used recently to understand the formation and thermodynamic properties of ice and clathrate hydrates at the nanoscale level^2^,^3^,^9^,^27^,^28^,^29^,^30^,^31^, yet excluding the mechanics of polycrystalline clathrate hydrates. For example, Zhao *et al*.^28^ reported the structures and phase behaviours of highly confined water, ice, amorphous ice and clathrate, in nano-dimensional space. Here, we report the mechanical instability of single-crystal and polycrystalline sI methane hydrates under both uniaxial depressurization and compression, the accommodation structures of cohesive grain boundaries of polycrystalline sI methane hydrates with a range of grain sizes and the destabilizing mechanisms elucidated by the massively parallel classic MD simulations. We first show the fracture behaviours of monocrystalline sI methane hydrates, the complicated multi-phase molecular structures of grain boundaries and maximum ultimate strength of polycrystalline gas hydrates at a critical grain size, representing a transition from compatible grain-boundary softening in fine-grained polycrystals to highly localized deformation initiated at stress-concentrating grain junctions in coarse-grained polycrystals. Our work also identifies that the plastic deformation of hydrate polycrystals must be accommodated by the competing dynamic processes of hydrate decomposition and reformation in the grain-boundary zone, revealing the intrinsic mechanism of deformation-induced hydrate dissociation, which was observed in the macro experiments. This finding suggests that sediment-hosted gas hydrates concentrating vast quantities of methane in nature could be further destabilized by geological tectonic, gravitational and anthropogenic force-induced ground deformation beyond the conventional thermodynamic instability.

## Results

### Brittle failure of monocrystalline sI methane hydrate

**Figure 1:**
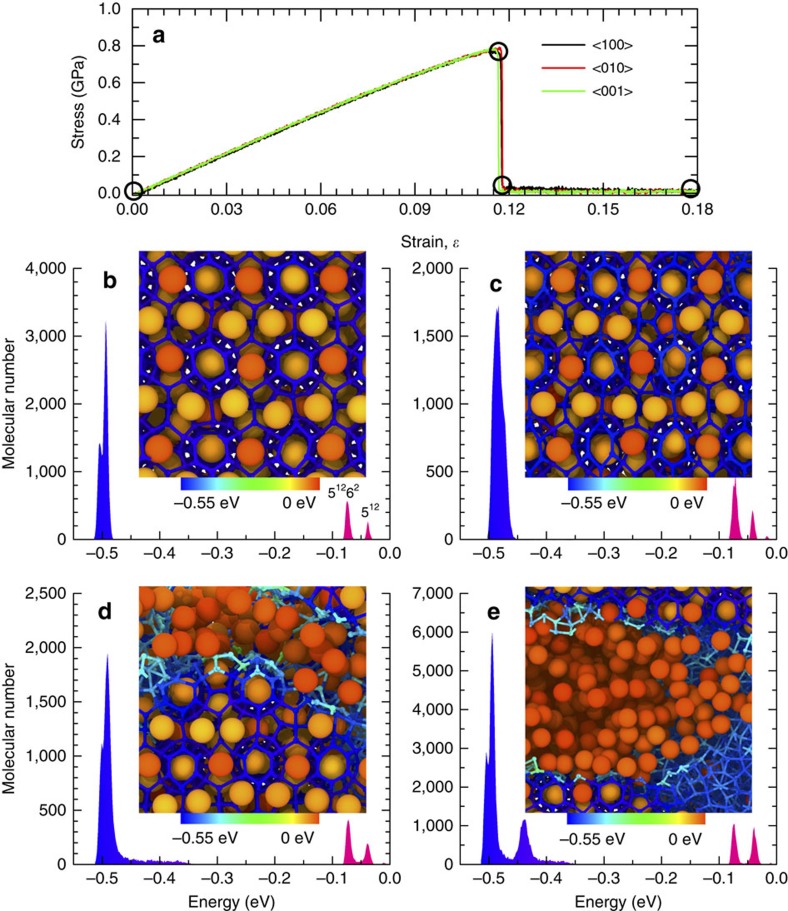
Mechanical response of single-crystal methane hydrate. (**a**) Stress–strain relationships for single-crystal methane hydrate. The molecular cohesive energy distributions and corresponding localized molecular structures (**b**) initially, (**c**) at onset of fracture, (**d**) immediately after initial fracture and (**e**) at a strain of 0.18.

Tension tests on an equilibrated monocrystalline sI methane hydrate with an 8 × 8 × 8 periodic supercell were first performed at 283.15 K and 10 MPa to investigate the hydrate's deformation behaviour. Both the large tetradecahedral (5^12^6^2^) and small dodecahedral (5^12^) polyhedral cages were fully occupied by methane. Figure 1a shows that an uniaxial loading along the three orthogonal directions yields overlapping stress–strain curves, implying elastically isotropic behaviour, consistent with the results of previous experiments and first-principles calculations^32^,^33^,^34^. The methane hydrates exhibit elastic behaviour followed by brittle failure, which is similar to the behaviour of water ice I_*h*_ (see Supplementary Fig. 1; Supplementary Note 1). A slight nonlinearity resulting from nonlinear elastic deformation occurs at high strain. The ultimate tensile stress occurs at a strain of ∼11.8%. The abrupt drop of the load indicates complete rupture. Depending on the temperature, the Young's modulus calculated from the initial elastic response up to 1% varies from 7.68 to 9.71 GPa (See Supplementary Figs 2 and 3), which agrees with available experimental and first-principles calculation results of 8.52 and 11.07 GPa, respectively^32^,^33^, as well as the Young's modulus reported for water ice (9.7–11.2 GPa) (ref. 35). These predictions were also verified by the most widely used atomistic models (SPC/E, TIP4P, TIP4P/2005 and TIP4P/ICE) and *ab initio* MD (AIMD) simulations (see Supplementary Figs 2–5; Supplementary Note 2). The typical structural developments of single crystals described by the MD simulation with the coarse-grained model, the SPC/E model, the TIP4P model and the AIMD simulation are shown in Supplementary Movies 1–4, respectively.

Figure 1b–e presents the potential energy distributions within the whole system for four distinct deformed stages marked by circles in Fig. 1a, together with their corresponding localized molecular structures. The water molecules of five- and six-membered rings are represented by two sharp peaks in the blue region (Fig. 1b). A close inspection of the cohesive energy per molecule reveals that the molecules situated in the six-membered rings of 5^12^6^2^ polyhedra have a slightly higher cohesive energy than those in the five-membered rings (see Supplementary Fig. 6). This might imply different mechanical stability of large and small water cages under deformation. Two peaks far from the left (approximately −0.04 and −0.07 eV) indicate the positions where methane molecules preferentially localize. This localization is depicted in the inset: the methane encapsulated in large-diameter 5^12^6^2^ cages is shown in yellow, whereas that trapped in 5^12^ cavities is shown in red. By comparing distribution function of the water potential energy in Fig. 1b,c, one can conclude that six-membered hexagonal rings undergo larger deformations than five-membered pentagonal rings. These various localized deformations imply that the fracture of sI methane hydrate will initiate at the hexagonal faces connecting larger 5^12^6^2^ cages into a cubic arrangement. The presence of a peak at the leftmost position in Fig. 1d,e signifies the imminent escape of methane from misshapen cages. Another new peak at approximately −0.44 eV in Fig. 1e corresponds to the fractured-cage water molecules. The cohesive energy of all water molecules in the large 5^12^6^2^ polyhedra increases during the deformation before the onset of fracture. The small variation of the methane cohesive energy shown in Fig. 1c originates from the molecular interactions between methane and water in the deformed cages: a strong repulsive force acts on methane in the transverse directions, whereas a weak repulsive force acts in the elongation direction. As evident in the insets of Fig. 1b–d, the geometry of the non-destructed 5^12^6^2^ cages changes from spherical to elliptical and then back to spherical. The inset of Fig. 1d also shows that the sI methane hydrate exhibits brittle-type fracture. This fracture results in the release of methane from damaged cavities and creates fresh surfaces. Similar to conventional solid materials, the water potential energy of cleaved surfaces of hydrate is ∼0.04 eV greater than that of their bulk counterparts (Supplementary Fig. 7). As shown in the inset of Fig. 1e, water molecules with broken hydrogen bonds spontaneously aggregate to form a liquid droplet that clings to the cleaved surfaces, whereas methane molecules are distributed in the interstice (Supplementary Figs 7–10; Supplementary Note 3).

### New grain-boundary structures of polycrystalline hydrates

**Figure 2:**
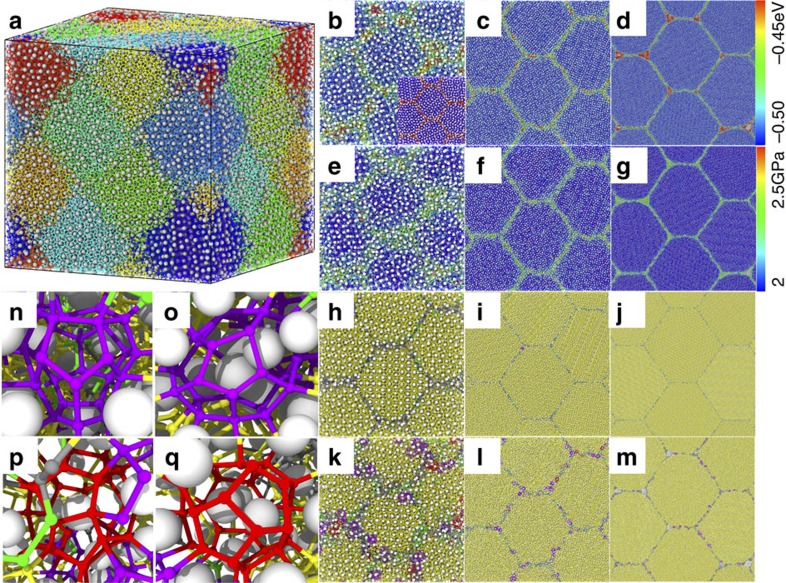
Molecular models of polycrystalline methane hydrates. (**a**) Three-dimensional polycrystals of methane hydrates with a grain size of 11.2 nm. Cross-sectional snapshots of polycrystals with grain sizes of (**b**) 5.61, (**c**) 15.0 and (**d**) 28.1 nm in their relaxed state. Molecules are rendered according to their potential energy. The inset of **b** represents the initial state. Cross-sectional snapshots of polycrystals with grain sizes of (**e**) 5.61, (**f**) 15.0 and (**g**) 28.1 nm. Molecules are coloured according to their von Mises stresses. Cross-sectional snapshots of polycrystals with grain sizes of (**h**) 5.61, (**i**) 15.0 and (**j**) 28.1 nm in their initial state. Water molecules are coloured according to their cage-type formation, whereas methane is coloured white. Cross-sectional snapshots of polycrystals with grain sizes of (**k**) 5.61, (**l**) 15.0 and (**m**) 28.1 nm in their relaxed state. Water molecules are coloured according to their cage-type formation, whereas methane is coloured white. The molecular structures of (**n**) 0CH_4_@5^12^6^3^, (**o**) 2CH_4_@5^12^6^3^, (**p**) 1CH_4_@5^12^6^4^ and (**q**) 2CH_4_@5^12^6^4^ were captured at the grain boundary.

Obtaining knowledge of grain-boundary structures is the first step in investigating the mechanical behaviours of polycrystalline of gas hydrates. However, such information is currently unavailable, and no laboratory measurements of the microstructural features of the grain boundaries of polycrystalline hydrates have been reported. We conducted MD simulations as a first attempt to elucidate the internal grain-boundary structures in polycrystalline methane hydrates. Figure 2a shows a representative three-dimensional molecular model of polycrystalline sI methane hydrates with submicron-sized grains, in which the water molecular cages are fully occupied by methane. The grains are illustrated by differently coloured water cages. Although the polycrystalline cells were initially cubic, they became slightly non-cubic during relaxation. Cross-sectional snapshots of three typically relaxed polycrystalline sI methane hydrates with grain sizes of 5.61, 15.0 and 28.1 nm are shown in Fig. 2b–g. The water molecules are rendered based on their potential energy and von Mises stress. The potential energy distribution in the initial polycrystalline structure shows that the grain boundary has a high energy level (inset of Fig. 2b). Despite the significant reduction of the grain-boundary energy by the relaxation process, water molecules at the grain boundaries have markedly higher potential energy and stress than those in the grain interior, comparable to the case of cleaved surfaces of monocrystalline methane hydrate. Meanwhile, the polycrystals with a grain size of 28.1 nm have a higher potential energy at the grain junctions than the polycrystals with a smaller grain size.

To better discern the molecular structure of the grain boundary, water molecules forming the cages in both the initial and relaxed systems were analysed. Figure 2h–m shows the analysed polycrystalline structures in both the initial and relaxed states, where a colour code was used (green: 5^12^ cages, yellow: 5^12^6^2^ cages, purple: 5^12^6^3^ cages, red: 5^12^6^4^ cages, grey: unidentified molecules and white: methane). Before relaxation, the water molecules in the grain interior are identified as the structural unit of sI methane hydrate (5^12^6^2^, 5^12^), whereas the unidentified water molecules at the grain–grain interfaces reveal the grain boundaries (Fig. 2h–j). Connections in some local area are observed between grains via either 5^12^6^2^ or 5^12^ cages due to the occasional lattice anastomosis of neighbouring grains. Figure 2k–m reveals that the relaxation of the polycrystalline structure significantly promotes microstructural rearrangement at energetically unfavourable grain boundaries. A comparison of Fig. 2h,k reveals that the width of the relaxed grain boundaries is slightly larger than those of the initial state because of molecular rearrangements at internal interfaces, whereas no changes in the grain interior are observed. However, the thickness of the grain boundaries is independent of the grain size. Numerous new uncommon 1CH_4_@5^12^6^3^, which have previously been suggested to facilitate the coexistence of typical sI and sII structures^2^, and few hexadecahedral single- and double-CH_4_@5^12^6^4^ are found to structurally bridge the grains of sI methane hydrates at the internal interfaces, reflecting the formation of cohesive grain-to-grain polycrystalline methane hydrates. This result is consistent with those of previous investigations showing that a polycrystalline methane hydrate solid can be achieved by sequences of sI and sII hydrates linked via layers with both 1CH_4_@5^12^6^4^ and 1CH_4_@5^12^6^3^ cages^36^. The small fraction of large 5^12^6^4^ cages confirms that the sII 5^12^6^4^ cages are too large to be stabilized by methane^19^. Interestingly, in addition to the detection of unusual 1CH_4_@5^12^6^3^, 0CH_4_@5^12^6^3^ and 1CH_4_@5^12^6^4^, previously unreported 2CH_4_@5^12^6^3^ and 2CH_4_@5^12^6^4^ structures are also observed in the grain boundaries of all the samples (Fig. 2n–q). In contrast to the 1 and 2CH_4_@5^12^6^4^ cages, both the 0CH_4_@5^12^6^3^ and 2CH_4_@5^12^6^3^ cages are greatly misshapen and therefore less stable. For polycrystals with a large grain size, tiny methane bubbles are detected at the grain junctions because of internal stress-induced dissociation, implying the difficulties in the laboratory preparation of high-quality methane hydrates. Previous cryogenic scanning electron microscopy imaging studies showed that synthetic polycrystalline methane hydrates have high intergranular porosity and sharply defined boundaries with water ice^37^. Overall, the predicted grain boundaries and grain junctions of polycrystalline methane hydrates are more sensitive, with a more complex molecular structure than those of conventional polycrystalline materials. Guest molecules encapsulated in the polyhedral cavities formed by host water molecules greatly affect the molecular-level structures of the grain boundary of polycrystalline hydrates; polycrystalline hydrates with low cage occupancy of guest methane molecules has a large number of unidentified cages (see Supplementary Fig. 11), implying lower stability of polycrystalline hydrate concentrating fewer methane.

### Ultimate strength of polycrystalline hydrates

**Figure 3:**
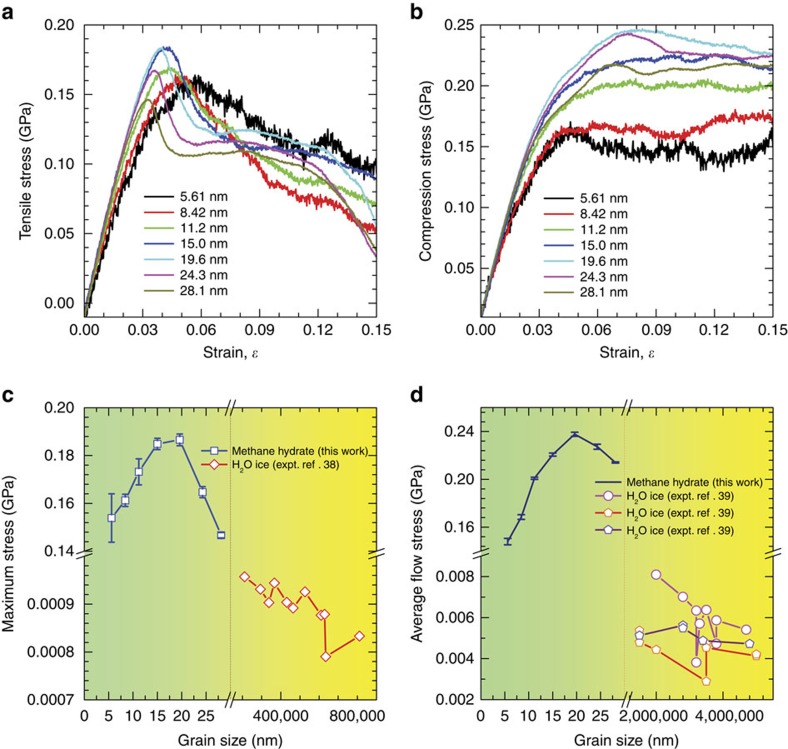
Mechanical properties of polycrystalline methane hydrates. Tensile (**a**) and compressive (**b**) stress–strain curves of polycrystals with grain sizes ranging from 5.61 to 28.1 nm. Tensile maximum stress (**c**) and average compressive flow stress (**d**) as a function of grain size, and the corresponding experimental value for water ice.

Irrespective of deformation mechanisms, grain size is known to play a central role in the apparent mechanical strength of conventional polycrystalline solid materials. Simulations of both uniaxial tensile (depressurization) and compressive loadings on polycrystalline methane hydrates were accordingly performed to determine the maximum ultimate strength. The resulting tension and compression stress–strain curves from 14 simulations with grain sizes that varied from 5.61 to 28.1 nm are plotted in Fig. 3a,b. Both curves are markedly different from that for the monocrystalline. Polycrystals apparently exhibit ductile characteristics, and the deformation process of polycrystals proceeds in three stages. First, instantaneous elastic stretching occurs. The loading responses subsequently become nonlinear as a result of the occurrence of coupled elastoplastic deformation instead of nonlinear elasticity. Finally, deformations under both loads are irreversible, but with different features. In the case of tension tests, all polycrystals exhibit an apparent strength maximum. The post-peak stress–strain relationship of polycrystals with a large grain size shows several distinctive stages of strain softening. Furthermore, even a slight strain-hardening behaviour occurs in the largest polycrystal after the first rapid strain softening. In contrast, under compressive loading, the polycrystals deform with a steady flow stress following monotonic strain hardening. The maximum tension and compression strengths are appreciably lower than those of perfect single-crystal methane hydrates. Figure 3c,d presents the maximum tensile stress and average compressive flow stress, which were determined at strain levels of 8–15% as a function of the grain size, as well as the corresponding values for macroscopic grain-textured polycrystalline water ice determined experimentally at low strain rate^38^,^39^. Intriguingly, the figures show a ‘flipped' behaviour of the maximum tensile and compressive flow stresses of polycrystalline methane hydrates; both increase as the grain size decreases to 15.0 and 19.6 nm, reaching ultimate values of ∼0.185 and 0.238 GPa, respectively, and then they decrease as the grain size decreases further. This transition of increasing to decreasing mechanical strength is reminiscent of the well-known Hall–Petch and inverse Hall–Petch effects of polycrystalline metals. The experimentally observed Hall–Petch behaviour of polycrystalline water ice arises from the operation of a Zener–Stroh cracking mechanism or intergranular cracking resulting from elastic anisotropy^39^. To examine whether the switching behaviour of mechanical strength might arise from the specific microstructure of hydrate polycrystals (uniformly shaped grains and identical grain size), additional simulations on the polycrystals with random geometrical grains were also carried out. Similar results were obtained, as shown in Supplementary Figs 12–14; Supplementary Movies 5 and 6).

**Figure 4:**
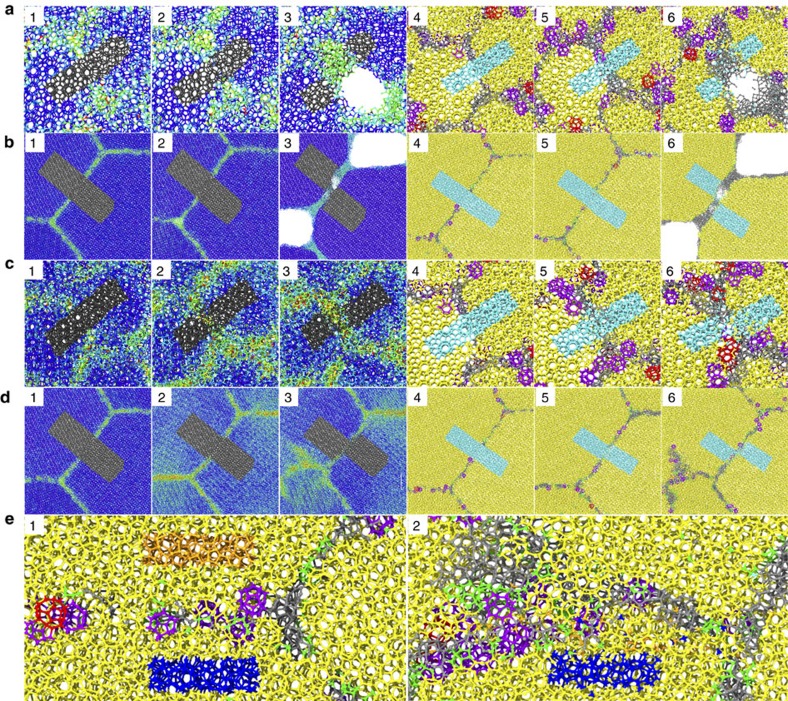
Typical localized grain-boundary structures under mechanical loading. Localized grain-boundary snapshots of polycrystalline methane hydrates with grain sizes of (**a**) 5.61 nm and (**b**) 28.1 nm at the initial, maximum stress and maximum preset strain stages under tension. Localized grain-boundary snapshots of polycrystals with grain sizes of (**c**) 5.61 nm and (**d**) 28.1 nm at strains of 0.0, 0.06 and 0.15 under compression. The snapshots of numbers 1–3 are coloured according to the molecular von Mises stresses, whereas the snapshots of numbers 4–6 are coloured according to their cage-type formation. (**e**) Zoomed-in molecular structures of polycrystals with a grain size of 28.1 nm at strains of 0.0 and 0.15, as framed in **d**4 and **d**6. Water particles are coloured with either aqua or black in **a**–**d** for monitoring grain-boundary sliding. Water particles are coloured with either blue or orange near the grain boundary in **e** for identifying the structural transformations upon pressurization.

To elucidate the deformation mechanisms behind the weakening and strengthening phenomena of polycrystalline methane hydrate, a molecular-scale analysis of the microstructural development of the polycrystals during loading was conducted. The aforementioned phenomena are attributed to the influence of grain size on the net grain-boundary accommodation deformation and hydrate phase change. Typical snapshots of the localized molecular structures of polycrystals coloured according to von Mises stress and type of clathrate cage are shown in Fig. 4. Before the peak stress, the elastic stretching of grains and grain-boundary recrystallization and dissociation dominate the response. As shown in Fig. 4a,c, more extensive metastable coexistence of 5^12^6^3^, 5^12^6^4^ cages and disordered water (see Supplementary Fig. 15 and Supplementary Movies 7–10), promoted by a large grain-boundary area, is the main source of the reductions of strength in polycrystalline sI methane hydrates with small grains, resulting in the inverse Hall–Petch effect. These reductions are analogous to those in polycrystalline metals and ceramics, where the large fraction of amorphous atoms located at the grain boundary facilitates their deformation, leading to the weakening phenomenon. The simulations show that the elastic grains of polycrystals are significantly stiffer than the grain boundaries for increasing loading (see Supplementary Movies 7–10). This distinct behaviour suggests that the deformation mainly results from local deformations at the grain boundaries and that deformation from the grains is negligible. The identical initial width and properties of the cohesive grain boundary indicate that large-grained polycrystals develop highly deformed cohesive zones along the grain boundaries. This behaviour is a result of the ability of large-grained polycrystals to generate higher stress concentrations than small-grained polycrystals.

For grains larger than 15 and 19.6 nm, the Hall–Petch type dependency of the strength of polycrystalline methane hydrates under both uniaxial tension and compression originates from an under-matched grain boundary and larger stress concentration at the grain junctions. However, for the case of polycrystalline metals, nucleation of dislocations in the grain interior destabilizes the polycrystals, resulting in a Hall–Petch type dependency for their mechanical strength. The mechanism underlying the Hall–Petch effect for polycrystalline methane hydrate is similar to that for polycrystalline graphene, in which larger stress concentrations occurring at the grain boundaries weaken the materials^40^. Under tension, the rapid strain-softening behaviour following the peaks is attributed to a cooperative effect of tilt grain-boundary sliding and intergranular decohesion (Fig. 4b). After the sliding and decohesion, elastic grains gradually restore the elastic strain and enhance the failure of the grain boundary. Grain-boundary sliding is the main source of the subsequent strain softening. During the sliding, cage destruction occurs at the deformed grain boundary, and the decohesion of the grain junction strongly dominates over its reformation, resulting in the formation of methane bubbles, bubble migration and coalescence and the reduction of the grain size (see Supplementary Movie 11). Augmentation of the strain markedly enhances depressurization at the grain junctions, leading to rapidly localized hydrate decomposition. This mechanism agrees with the simulation results of the macroscopic finite element method^41^. The gas accumulation and capillary suction allows the dissociated water and gas to access the grain boundaries, weakening them. This enhances the boundary sliding and leads to the premature tensile failure of large polycrystals. However, upon compression, a palpable solid-state structural transformation occurs (see Supplementary Movie 12). Both experiments and simulations have shown that gas hydrates tend to undergo structural phase transitions under applied pressure^42^,^43^,^44^,^45^. The combination of the structural transformation and grain-boundary sliding explains the pronounced steady flow stress. For the largest grain size, compressive stress-induced hydrate dissociation ultimately nucleates and propagates into the grain interior (Fig. 4d). This deformation-induced dissociation behaviour of polycrystalline methane hydrates was also observed in X-ray diffraction analyses of pre- and post-deformed methane hydrate polycrystals as well as in rheology experiments^20^,^21^,^22^,^23^. In the experiments, decomposed water was frozen into water ice because of the sufficiently long reaction time used, whereas the fresh water from decomposed hydrates has a disordered arrangement in our nanosecond simulations.

Unlike the compacting of dense single-component water ice, deformed grains of methane hydrates do not undergo dislocation. This lack of dislocation might be due to the encaged methane constraining the cooperative mobility of the cage-structured water molecules. Monitoring of the orange-highlighted water cages near the grain boundary reveal that they decompose because of compressive stress concentration, forming a newly curved grain boundary (Fig. 4e; see Supplementary Movie 13). Most of the hydrate-decomposed water diffuses across the new dynamic grain boundary and reforms clathrate cages. In addition, the blue-coloured water cages at adjacent grains reveal that the small amount of water that migrated from its original lattice zone formed fresh clathrate cages. This finding provides visual confirmations of the mobility of water molecules in the host lattices of clathrate hydrates, in agreement with previous experimental findings^46^.

## Discussion

**Figure 5:**
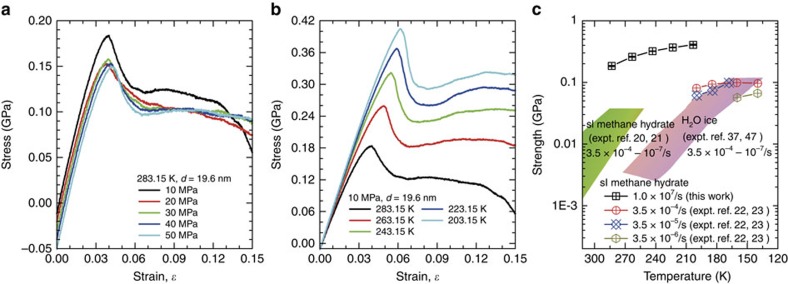
Mechanical properties of polycrystalline methane hydrates under different external conditions. Stress–strain relationships for polycrystalline hydrates with a grain size of 19.6 nm at (**a**) 283.15 K and various confining pressures from 10 to 50 MPa, and at (**b**) a confining pressure of 10 MPa and temperatures ranging from 203.15 to 283.15 K. (**c**) Maximum strength of polycrystals as a function of temperature, as well as the corresponding experimental data for both water ice and methane clathrate^20^,^21^,^37^,^47^ measured at strain rates of 3.5 × 10^−4^ to 3.5 × 10^−7^.

The stability of methane hydrates is extremely susceptible to changes in their cage occupancy (see Supplementary Fig. 16; Supplementary Note 4) and environmental conditions, such as their temperature and confining pressure. A series of simulations of elongation behaviour of a selected polycrystal with a grain size of 19.6 nm under confining pressure and temperature ranging from 203.15 to 283.15 K and from 10 to 50 MPa were carried out. Figure 5a,b presents the resulting stress–strain curves as a function of confining pressure and temperature, and Fig. 5c compares the mechanical strength with available experimental data^20^,^21^,^22^,^23^,^37^,^47^. All of the stress–strain curves include a linear component; however, they subsequently become nonlinear. The Young's modulus (∼6.28 GPa) is found to be independent of confining pressure and ∼20% lower than that of single crystals. A lower Young's modulus is caused by the large fraction of molecules in the grain boundaries, similar to the case of other polycrystalline materials. In addition, Fig. 5a also reveals that, when the confining pressure exceeds 10 MPa, an unexpected increase of the confining pressure does not lead to enhancement of mechanical strength at the given temperature of 283.15 K. Experimentally, triaxial compression measurements of artificial methane hydrate demonstrated a transition from strengthening to weakening at a critical confining pressure of 10 MPa (ref. 48). At pressures below 10 MPa, the strength monotonically increases with an increase in confining pressure, whereas it decreases slightly as the confining pressure increases above 10 MPa. This behaviour indicates that a confining pressure limitation exists for enhancing the grain-to-grain coordination and friction resistance. As observed in Fig. 5b,c, the mechanical strength and Young's modulus are strongly temperature dependent, where a higher Young's modulus and greater strength are observed at lower temperatures. This finding confirms that the strength increases with decreasing temperature, as observed in experiments^20^,^23^,^48^. In comparison, the significant discrepancy in the calculated and experimentally observed strengths stems mainly from the differences of several orders of magnitude in strain rate and grain size and from impurities and pores in the experimental samples. After the rapid strain softening, a high confining pressure results in almost constant tensile flow stress, whereas methane hydrate exhibits a transition from strain softening to apparent strain hardening at low temperatures. A high confining pressure is unable to enhance the grain-to-grain resistance, whereas low-temperature conditions restrict grain-to-grain decohesion and the deformation of polycrystals.

In contrast to the grain boundaries of polycrystalline metals, the cohesive grain boundaries of polycrystalline sI methane hydrates are represented by complex multi-phase molecular structures composed of sI, uncommon metastable pentakaidecahedral and hexadecahedral cages, and disordered water structures. These structures facilitate the development of the polycrystalline clathrate hydrate growth mechanism. Both tension and compression tests of polycrystalline methane hydrates show that their mechanical stability is strongly grain-size and grain-morphology dependent in ways not previously identified. This dependence is attributed to the influence of grain size on the net grain-boundary deformation. Monocrystalline methane hydrates fail in a brittle manner, whereas the plastic deformation of polycrystals must be accommodated by the competing dynamic processes of hydrate decomposition and reformation in the grain-boundary zone. These competing processes include the formation of bubbles, bubble migration and coalescence, heterogeneous nucleation, lattice–water diffusion and grain-boundary sliding, all of which impair the mechanical strength of polycrystalline gas hydrates and their bearing sediments. Our findings provide a complete molecular elucidation of the strain-induced structural transformation and hydrate dissociation observed in deformation experiments of synthetic polycrystalline methane hydrates^20^,^22^,^23^.

Naturally occurring and synthetic gas hydrates with micropores and grains of several tens or hundreds of micrometres in size are beyond the capacity of classic MD simulations. However, our MD simulation results show a Hall–Petch effect in polycrystalline methane hydrates when the grains exceed a critical size. This Hall–Petch effect is also experimentally observed in coarse-grained polycrystalline ice^38^, implying an even lower mechanical strength of both natural and artificial hydrate with micrometre-sized grains. Strain-induced hydrate dissociation and grain-boundary sliding of pure polycrystalline sI methane hydrate indicate that the dissociation of natural gas hydrates even in their thermodynamic stability field can also be triggered by the inevitable ground deformation caused by geological tectonic, gravitational and anthropogenic forces, such as earthquakes, storms, sea-level fluctuations or man-made disturbances (including well drilling and gas production from hydrate reservoirs). Such ground deformation will also decrease the effective stress, cohesion and frictional resistance between sediment grains, especially for sediments in which solid hydrates function as cement or a support framework^8^. Our findings have direct implications for understanding the stability, safety and evolution scenarios of gas hydrate reservoirs and global climate change and geohazards associated with massive hydrate dissociation in both terrestrial and planetological environments^6^,^10^,^11^,^12^,^13^,^14^,^16^,^17^,^20^,^22^.

## Methods

### Single crystals

The starting position of the oxygen atoms and the centres of mass of the methane molecules were taken from the X-ray diffraction analysis results for ethylene oxide hydrate reported by McMullan and Jeffrey^49^. The Initial hydrogen atoms of water molecules within one unit crystal cell were randomly assigned, and a short Monte Carlo integration was performed to generate the lowest-energy proton-ordered H-bonded network, in agreement with the Bernal–Fowler rule. The mechanical responses of single crystals methane hydrate constructed by this procedure are similar to that with a zero net dipole moment configuration prepared by Takeuchi *et al*.^50^

### Polycrystals

Two distinct textured microstructures consisting of sequences of sI hydrate grains were chosen. For the first microstructure, seven polycrystals were constructed with dimensions ranging from 14.1 × 14.1 × 14.1 to 70.7 × 70.7 × 70.7 nm^3^ based on a Voronoi construction^51^. Initially, seeds with ordered body-centred cubic arrangement sites were placed in a three-dimensional supercell equal to that of the monocrystalline sI methane hydrate sample. Second, one copy of the original configuration of methane hydrate with periodic boundary conditions was rotated around the seed, and then cut out by a truncated octahedron around the seed. Finally, all the as-cut truncated octahedron grains were assembled to form a polycrystal with dimensions identical to that of the single-crystal sample. To avoid artificial molecule (monatomic particle) overlaps in the polycrystals, molecules protruding beyond the grain boundaries were deleted when part of a molecule pair was with a nearest-neighbour distance of less than 0.1 nm. The resulting grain possesses the geometry of the body-centred cubic Wigner–Seitz cell, which is a truncated, octahedral, 14-faced Archimedean solid with faces 8 {6}+6 {4} (that is, eight regular hexagonal plus six square faces). Each polycrystal contained 16 identically sized grains of random orientation, as shown in Fig. 2a. The as-constructed samples with Voronoi grains size ranging from 5.61 to 28.1 nm contained ∼93,000 to 11,700,000 total water and methane molecules.

For the second microstructure, nine samples of fixed molecules of Voronoi grains with random geometry were assembled into a cubic structure with an edge length of 58.9 nm. This joining resulted in grain sizes that varied from ∼5.90 to 29.5 nm. The total number of molecules was ∼6,750,000, containing 8–512 randomly orientated grains. Specifically, a fully atomic model of polycrystalline sI methane hydrate with grain size of around 6 nm was created to contrast the coarse-grained models of polycrystals (Supplementary Figs 17 and 18).

The different cases produced polycrystals whose grain-boundary and grain-junction areas and grain roundness differed. Their grains were randomly crystallographically oriented. Previous powder X-ray analyses of laboratory-prepared methane hydrate samples did not detect a preferred crystallographic orientation of polycrystalline grains^23^. Periodic boundary conditions were used to obtain data for the thermodynamic limits.

### Forcefields

Both the host (water) and guest (methane) molecules were described using the monatomic model, which represents each molecule as a single sphere. The Stillinger–Weber model for the gas hydrate was used to model the tetrahedral short-ranged interaction potentials of monatomic water and those of the water–methane and methane–methane interactions^52^,^53^. Such a coarse-grained molecular model of water and methane is more than twice as efficient as a fully atomistic model in reproducing a range of properties of the liquid and solid phases of water and methane^2^,^3^,^52^,^53^,^54^,^55^,^56^. To verify the performance of this coarse-grained model in the context of mechanical responses, four popular atomistic non-polarizable water interaction models (SPC/E, TIP4P, TIP4P/2005 and TIP4P/ICE) and AIMD simulations were used. For these atomistic models, the intermolecular interaction potential was a Lennard-Jones site–site potential plus interatomic Coulombic interaction. The DACNIS united-atom model^57^ and fully atomic Optimized Potentials for Liquid Simulations model^58^ were adopted to describe methane. Unlike-pair interactions followed the standard Lorentz–Berthelot combination rule. The details of the classic atomistic simulations with fully atomic models and the AIMD simulation are presented in the Supplementary Figs 2–5 and 17–20 and Supplementary Notes 2 and 5.

### Simulations

Before the MD simulations of uniaxial loading, the monocrystalline and polycrystalline sI methane hydrates were quasi-statically relaxed to a local minimum energy configuration via the Polak–Ribiere version of conjugate gradient method^59^ with an energy tolerance of 1.0 × 10^−0^ eV and a force tolerance of 1.0 × 10^−0^ eV Å^−1^. The relaxation was performed with a simulation time of 10 ns under cold (*T*=283.15 K) and pressurized (*p*=10 MPa) hydrate-forming conditions in the *NpT* ensemble (constant number of particles, constant pressure and constant temperature) using the Nosé–Hoover barostat and thermostat with damping time constant *τ*_T_=2 ps and *τ*_p_=10 ps, which allowed unfavourable configurations in the grain boundaries to relax. This process can be observed by comparing Fig. 2h,k. The r-RESPA multiple-timestep algorithm was used to integrate the equations of motion with shorter and longer time steps of 10 and 20 fs (see Supplementary Fig. 21). The uniaxial loading was simulated by the deformation-control technique. The procedure was carried out on the relaxed polycrystalline models with a reasonable constant strain rate of 10^7^ s^−1^ by uniformly rescaling the *z*-coordinates of all atoms every 100 time steps. This deformation simulation set-up corresponds to a modified *NpT* ensemble, specifically, *NVT* in the loading direction, and N*p*T in the lateral directions. The relaxation MD steps were integrated in *NL*_*z*_*p*_*x*_*p*_*y*_*T* (*L*_*z*_ represents the length of simulation box along the tension direction) ensemble with a Nosé–Hoover anisotropic barostat and thermostat, which controls pressure only in *x* and *y* directions independently and temperature. This *NZ*_*z*_*p*_*x*_*p*_*y*_*T* ensemble with a Nosé–Hoover barostat and thermostat allows the polycrystalline structures to experience expansion/contraction in the transverse directions as a result of the Poisson effect. A total strain of 15% was predefined in the calculations. A stress-control technique with identical average strain rate produced a very similar mechanical response in the elastic region with deformation-control loading (see Supplementary Fig. 22 and Supplementary Note 6). The atomic stress per atom was calculated according to the virial definition of stress, using the forces on the atoms collected during the MD process. As is customary, both the output atomic potential energy and von Mises stress were averaged over 1,000 time steps to eliminate the fast oscillations. The mechanical stress by summing up the viral stress was nearly equivalent to that by using force-across-a-plane/area of that plane during a uniaxial deformation in the elastic domain (see Supplementary Figs 23 and 24). In addition to the calculation of the potential energy and stress of water, four water molecular cages were identified (—specifically, 5^12^6^*n*^ with *n*=0, 2, 3 and 4) based on the connectivity of the water molecules and the topology of the water rings, as introduced by Jacobson *et al*.^3^,^54^,^55^ The identification of these cages facilitated the analysis of the grain-boundary structure of the polycrystalline methane hydrates. All the calculations were performed by using the Large-scale Atomic-Molecular Massively Parallel Simulator^60^ software package on the Vilje SGI Altix 8600 computer cluster consisting of 1,440 nodes interconnected with a high-bandwidth low-latency switch network (FDR Infiniband) at Norwegian University of Science and Technology (Trondheim, Norway).

## Additional information

**How to cite this article:** Wu, J. *et al*. Mechanical instability of monocrystalline and polycrystalline methane hydrates. *Nat. Commun.* 6:8743 doi: 10.1038/ncomms9743 (2015).

## Supplementary Material

Supplementary Information Supplementary Figures 1-24, Supplementary Notes 1-6 and Supplementary References

Supplementary Movie 1 Visualisation of deforming single-crystal methane hydrates trajectory at 283.15 K and 10 MPa. Coarse-grained model was utilized for description of methane hydrates. Molecular particles are coloured according to its potential energy.

Supplementary Movie 2 Visualisation of deforming single-crystal methane hydrates trajectory at 1 K and 10 MPa. The SPC/E was employed to model methane hydrate. Grey line represents the hydrogen-bonds between waters. Atoms are coloured according to its potential energy.

Supplementary Movie 3 Visualisation of deforming single-crystal methane hydrates trajectory at 1 K and 10 MPa. The TIP4P was employed to model methane hydrate. Grey line represents the hydrogen-bonds between waters. Atoms are coloured according to its potential energy.

Supplementary Movie 4 Deformation of single-crystal methane hydrates trajectory of ab-initio molecular dynamic (AIMD) simulation at 283.15 K and 10 MPa. Blue sticks denote the hydrogen bonding.

Supplementary Movie 5 Animation of polycrystalline methane hydrates with uniformly shaped grains and grain size of 28.1 nm under tension strain. Water particles are coloured based on its potential energy.

Supplementary Movie 6 Animation of polycrystalline methane hydrates with randomly shaped grains and average grain size of 25.8 nm under tension strain. Water particles are coloured based on its potential energy.

Supplementary Movie 7 Animation of localised polycrystalline methane hydrates with uniformly shaped grains and grain size of 5.61 nm under tension strain. Except that specific water particles are black-painted for monitoring the grain-boundary sliding, water particles are coloured based on its von Mises stress. Methane particles are not shown for clarification.

Supplementary Movie 8 Animation of localised polycrystalline methane hydrates with uniformly shaped grains and grain size of 5.61 nm under tension strain. Except that specific water particles are aqua-painted for monitoring the grain-boundary sliding, water particles are coloured based on its cage type of formation. Methane particles are not shown for clarification.

Supplementary Movie 9 Animation of localised polycrystalline methane hydrates with uniformly shaped grains and grain size of 5.61 nm under compression strain. Except that specific water particles are black-painted for monitoring the grain-boundary sliding, water particles are coloured based on its von Mises stress. Methane particles are not shown for clarification.

Supplementary Movie 10 Animation of localised polycrystalline methane hydrates with uniformly shaped grains and grain size of 5.61 nm under compression strain. Except that specific water particles are aqua-painted for monitoring the grain-boundary sliding, water particles are coloured based on its cage type of formation. Methane particles are not shown for clarification.

Supplementary Movie 11 Animation of localised polycrystalline methane hydrates with uniformly shaped grains and grain size of 28.1 nm under tension strain. Except that specific water particles are aqua-painted for monitoring the grain-boundary sliding, water particles are coloured based on its cage type of formation. Methane particles are not shown for clarification.

Supplementary Movie 12 Animation of localised polycrystalline methane hydrates with uniformly shaped grains and grain size of 28.1 nm under compression strain. Except that specific water particles are aqua-painted for monitoring the grain-boundary sliding, water particles are coloured based on its cage type of formation. Methane particles are not shown for clarification.

Supplementary Movie 13 Animation of grain-boundary of polycrystal with uniformly shaped grains and grain size of 28.1 nm under compression strain. Except that specific water particles closed to the grain-boundary are orange- and blue-painted for monitoring the decomposition and solid-state structural transformation of grain-boundary, water particles are coloured based on its cage type of formation. Methane particles are not shown for clarification.
