# Supplementary material for: Mechanical instability of monocrystalline and polycrystalline methane hydrates
Source: Nat Commun. 2015 Nov 2;6:8743. doi: 10.1038/ncomms9743 (PMC4659946; doi:10.1038/ncomms9743)
Supplement: Supplementary Information — Supplementary Figures 1-24, Supplementary Notes 1-6 and Supplementary References [file ncomms9743-s1.pdf]

Single-crystal Ice-I$_h$ VS single-crystal methane hydrate

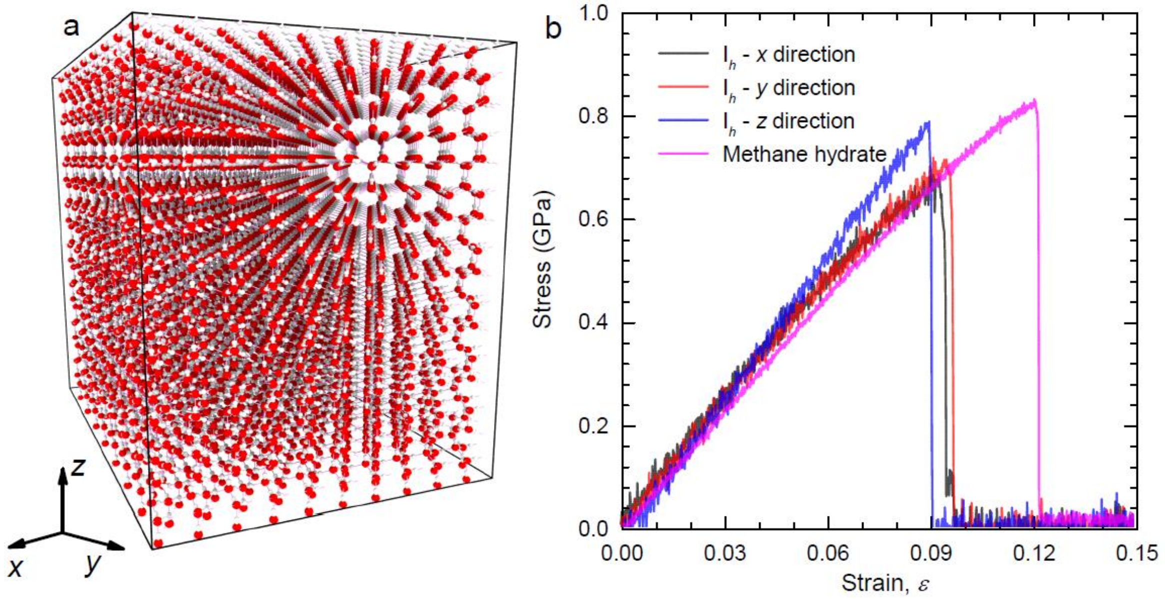

Supplementary Figure 1 | Single-crystal Ice-I$_h$ VS single-crystal methane hydrate by coarse-grained model (a) perspective model of single-crystal Ice-I$_h$ with $10 \times 8 \times 8$ super unit-cells and **(b)** a comparison of stress-strain curves of single-crystal Ice-I$_h$ and methane hydrate by coarse-grained model.

Coarse-grained model VS atomistic models at low temperature

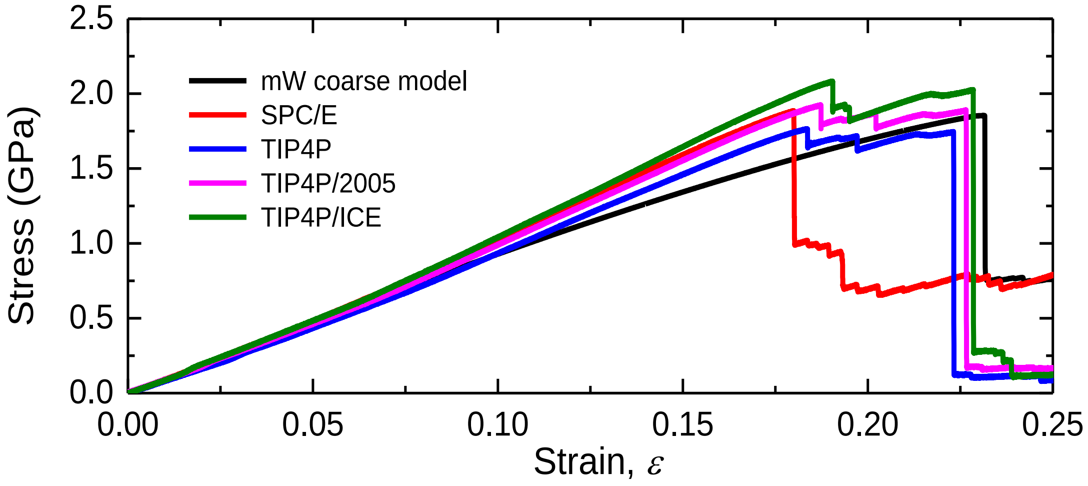

Supplementary Figure 2 | Comparison of stress-strain relation for five different models at extremely low temrpature of 1 K and confining pressure of 10 MPa. A low temperature is one important factor for the stabilization of methane hydrate. Deforming this icy material at extremely low temperature might allow one to observe different deformational response. Similarly, all the samples exhibit a long-range elastic deformation. A slight strain-hardening is observed after certain strain for the case of four atomistic models. Posterior to the first drop of tension stress, several drops of tension stress are also observed in late straining stage.

Coarse-grained model VS atomistic models at different temperatures

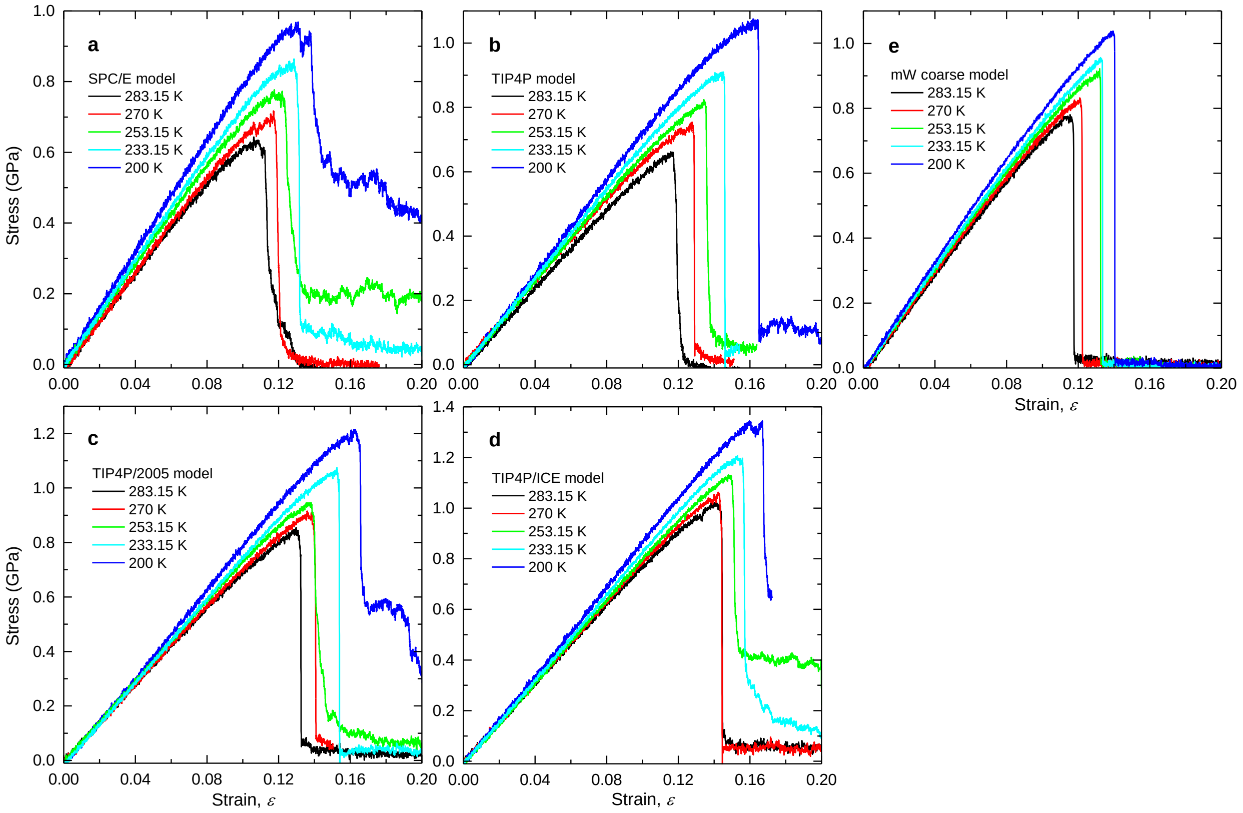

Supplementary Figure 3 | Tensile stress-strain responses of single crystal methane hydrate at a constant confining pressure of 10 MPa and temperature from 200 to 283.15 K. The single crystal methane hydrate with (a) SPC/E, **(b)** TIP4P, **(c)** TIP4P/2005, **(d)** TIP4P/ICE, **(e)** Coarse-grained models. All models predict an elastic deformation behavior of single crystal methane hydrate and catastrophic fracture without yielding in this temperature range, similar to that of water ice, see Supplementary Figure 1. As is expected, the value of mechanical parameters of methane hydrate such as Young's modulus, ultimate tensile stress, and critical strain, is inversely proportional to the temperatures.

Coarse-grained model VS atomistic models in hydrogen-bonds per unit cell

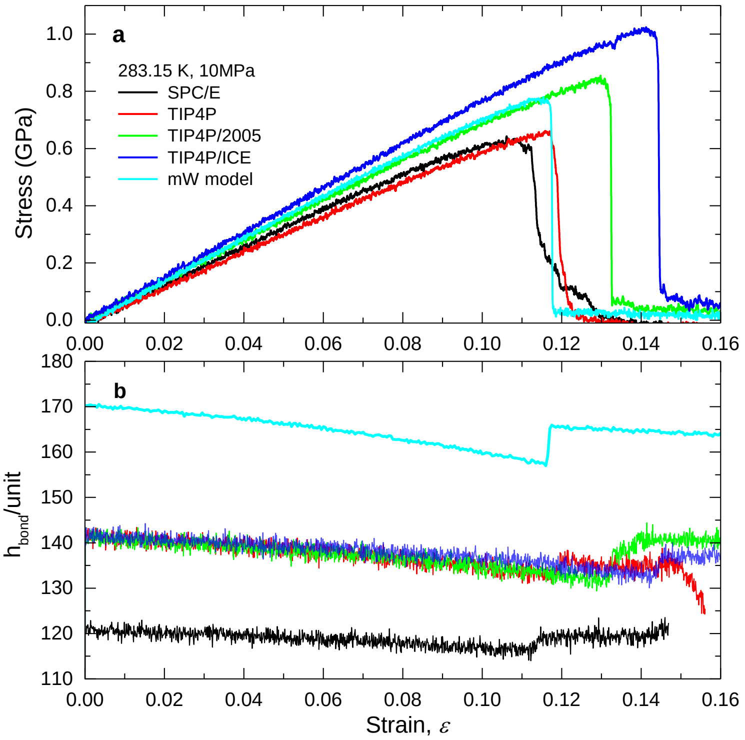

Supplementary Figure 4 | Stress-curves and variation in hydrogen-bonds per unit cell of single crystal methane hydrate with five different models during the straining at a constant confining pressure of 10 MPa and specific temperature of 283.15 K. (a) stress and **(b)** number of hydrogen-bonds per unit cell agaist the strain. The failure of methane hydrate comes directly from the breakage of the hydrogen-bonded networks. From Supplementary Figs.2-4, we conclude that the coarse grain model gives an average representation of the mechanical behavior of single-crystal methane hydrate, and shall therefore continue to use this model.

***ab initio* molecular dynamics (AIMD) simulation**

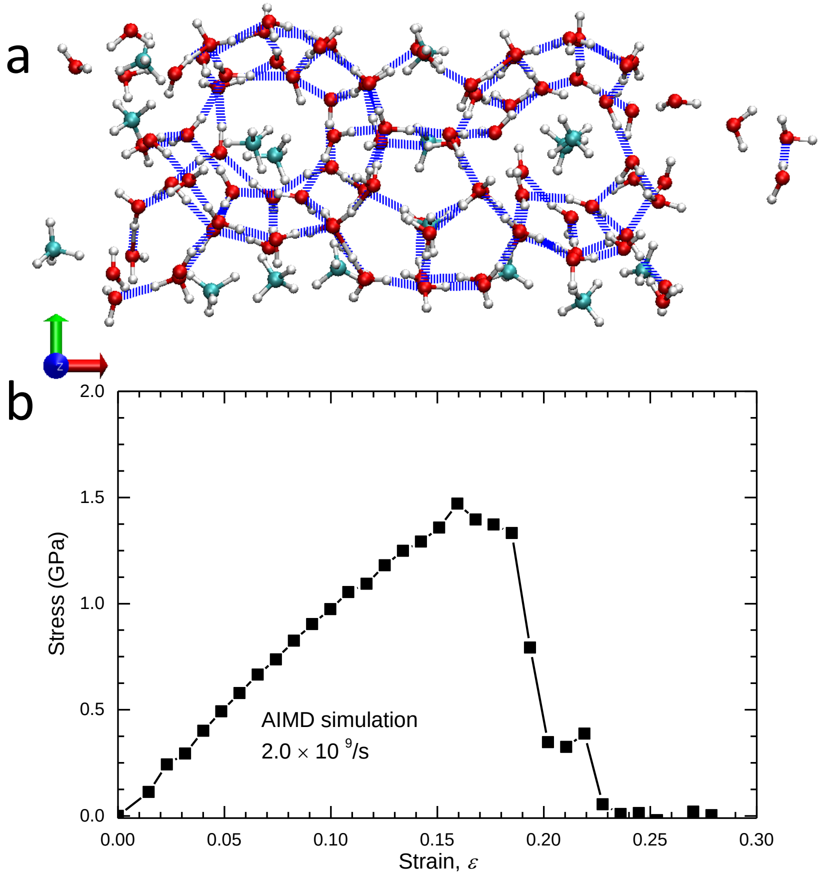

Supplementary Figure 5 | Results of AIMD simulations. (a) snapshot of fractured monocrystalline methane hydrate at strain of around 0.19. **(b)** stress-strain relation of single crystal methane hydrate through AIMD simulations at temrpature of 283.15 K and confining pressure of 10 MPa. Hydrogen bondings are represented by the blue spring sticks.

Water potential energy of small and large cages

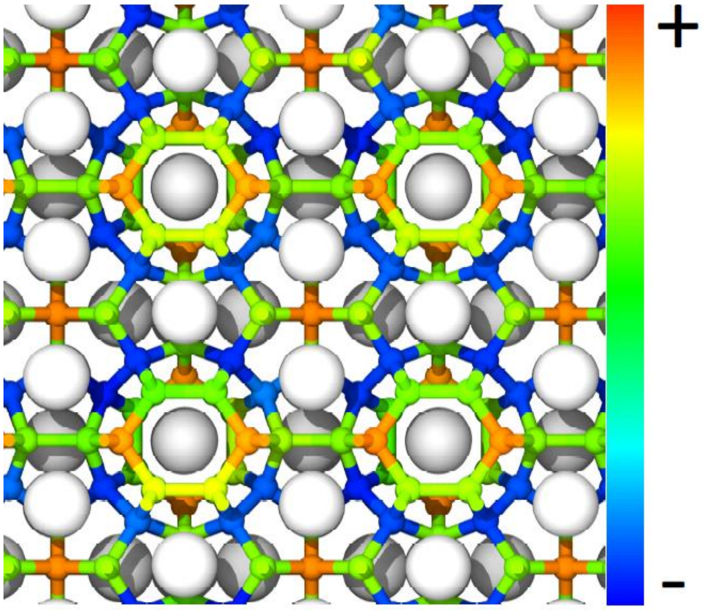

Supplementary Figure 6 | Localised single-crystal methane hydrate motif. Colouring of water is based on its pontential energy. Methane which occupys both 5^{12} and $5^{12}6^{2}$ nano-cages are white-painted. Water forming six-membered rings possesses larger potential energy than those forming five-membered rings, confirming the presence of two peaks in Fig. 1b of main manuscript.

Water potential energy of cleaved surfaces

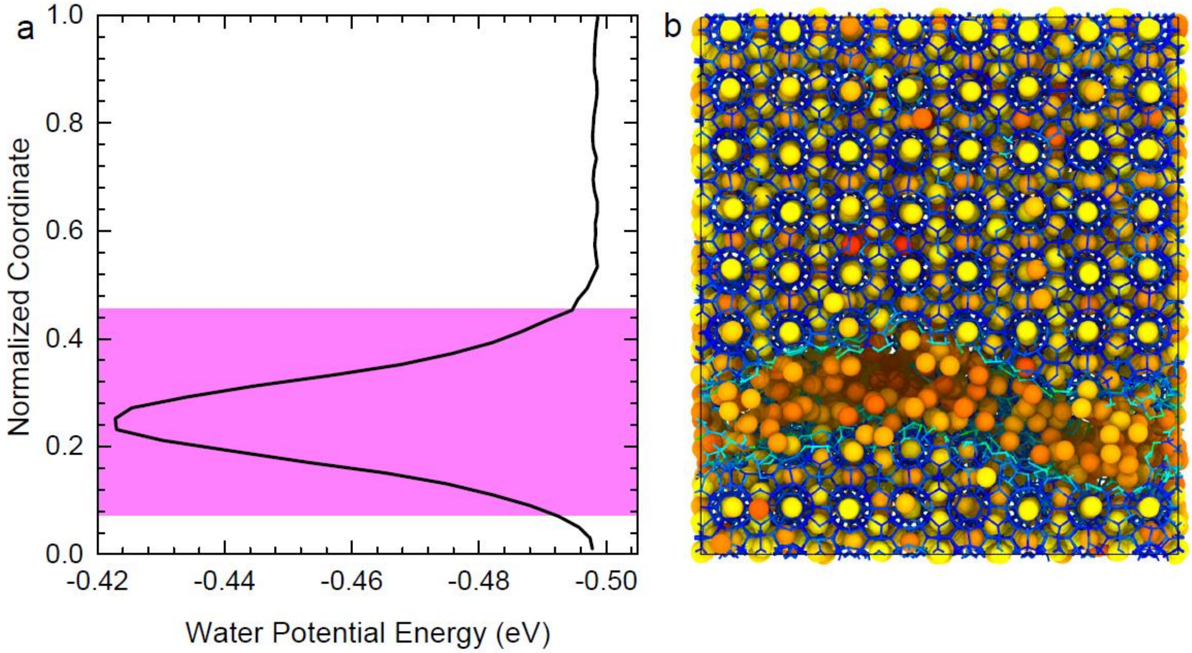

Supplementary Figure 7 | Water potential energy of cleaved surfaces. (a) distribution profile of water potential energy of fractured monocrystalline methane hydrate at strain of around 12%. **(b)** snapshot of a fractured monocrystalline methane hydrate. It is readily observed that potential energy of water stayed at the cracked region is higher than that of the bulk, particularly for the water in the interstice of fractured methane hydrate. Similar to conventional solid materials, the potential energy of cleaved surfaces of hydrate is approximately 0.04 eV higher than their bulk counterparts.

Average projected distribution profile of methane particles

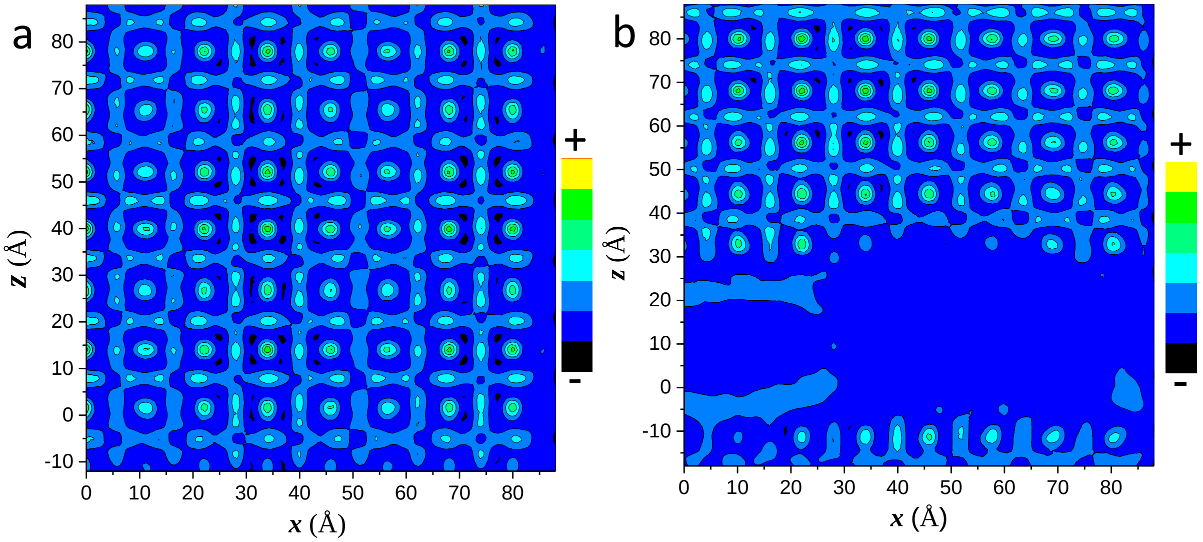

Supplementary Figure 8 | Average projected distribution profile of methane particles in the deformed monocrystalline methane hydrate (a) before and **(b)** after facture.

Distribution profile of methane particles

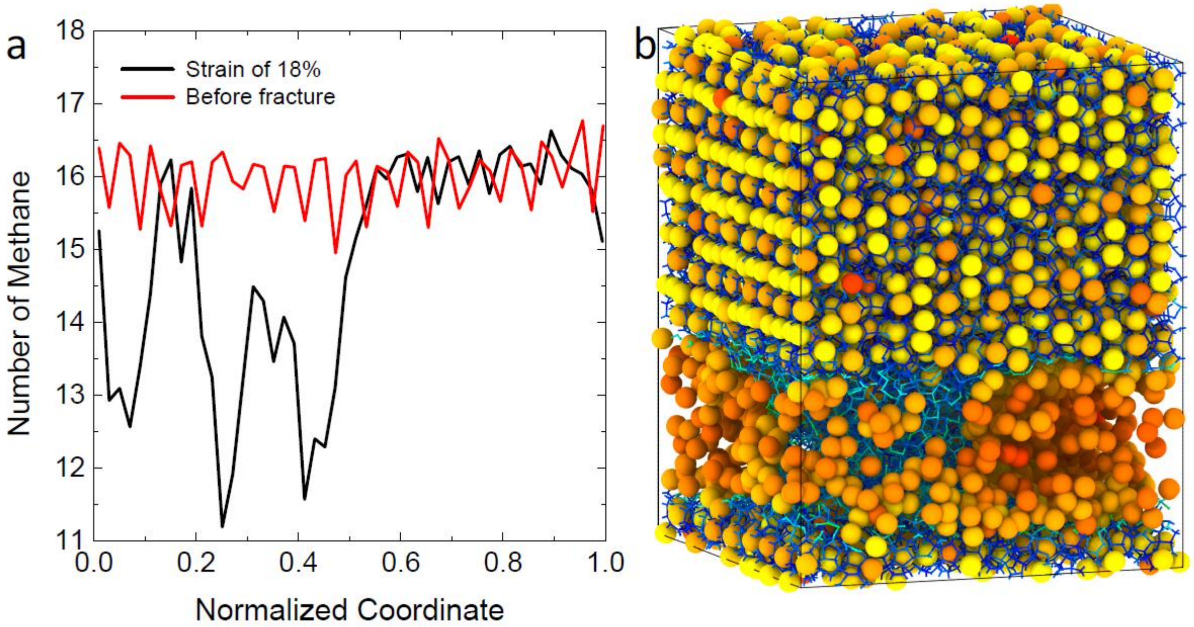

Supplementary Figure 9 | Distribution profile of methane particles. (a) distribution profile of methane particles in the monocrystalline methane hydrate at strain of 0 and 18%. **(b)** snapshot of molecular structure of monocrystalline methane hydrate at strain of 18%.

MSDs of water and methane molecules in methane hydrates

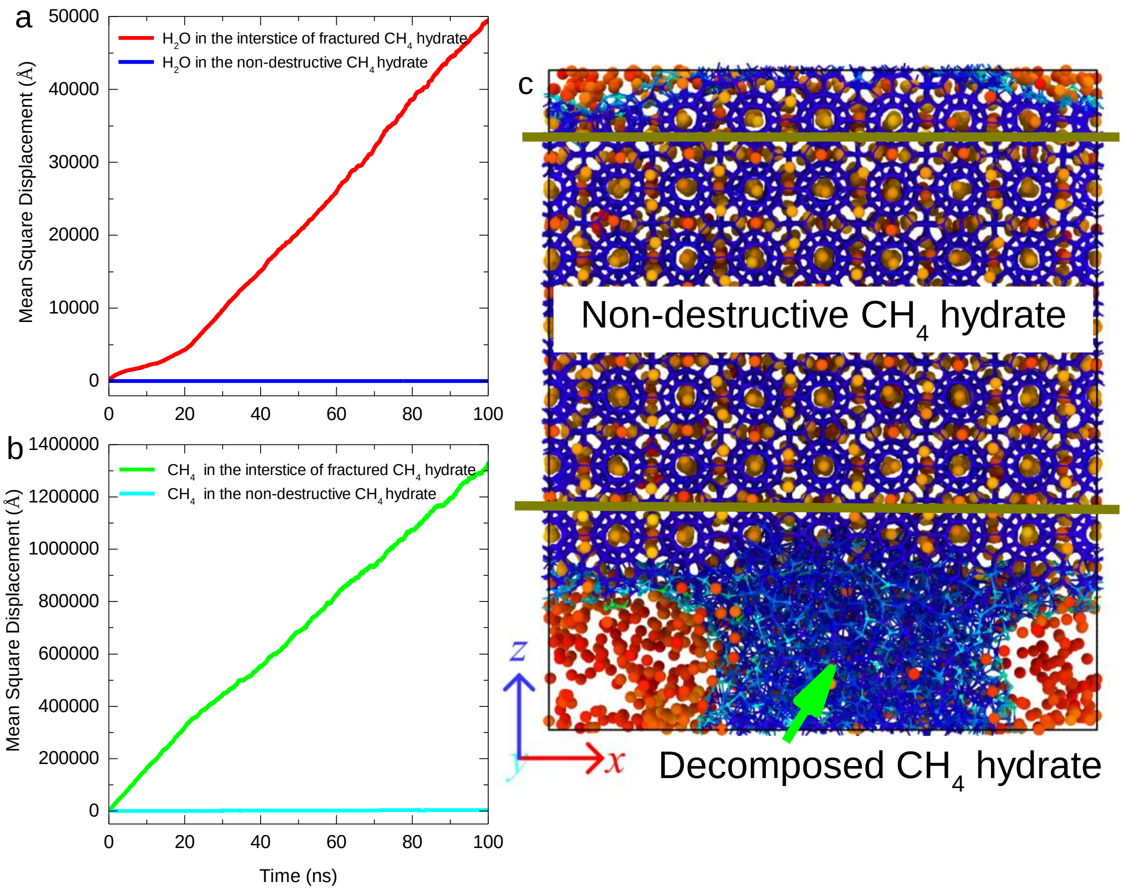

Supplementary Figure 10 | Mean square displacements (MSDs) of (a) water and **(b)** methane molecules in the interstice of fractured hydrate and in the non-destructive hydrate, as well as **(c)** a snapshot of fractured methane hydrate.

Effect of cage occupancy on the grain boundary microstructures

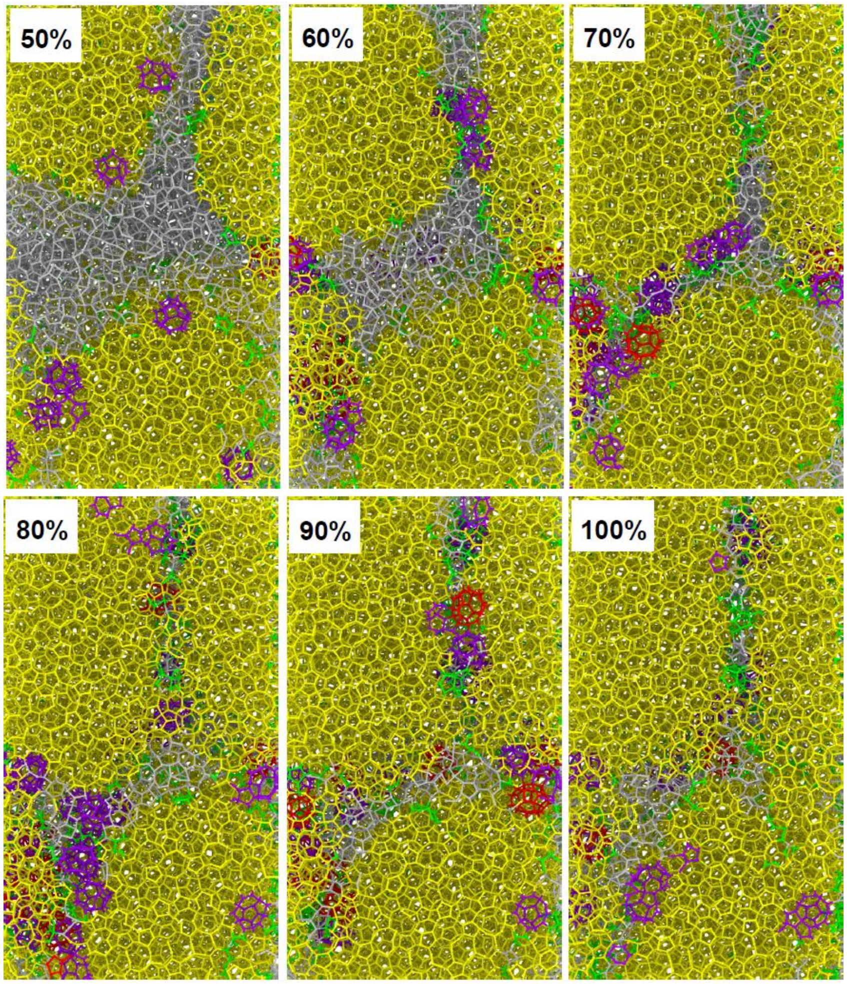

Supplementary Figure 11 | Local grain boundary structures of polycrystalline hydrates with methane occupation from 50-100%. The methane molecules were removed for clarification and the water-cages were coloured according to the type of cages (Green: 5^{12} cages, Yellow: $5^{12}6^{2}$ cages, Purple: $5^{12}6^{3}$ cages, Red: $5^{12}6^{4}$ cages, Grey: unidentified cages). It is readily observed that methane molecules encapsulated in the polyhedral cavities formed by a host water lattice greatly affects the molecular-level structures of the grain boundary of polycrystalline hydrates; the polycrystalline hydrates with low cage-occupancy of guest methane molecules has a large number of unidentified cages. This corresponds to a low mechanical stability of polycrystaline hydrates with low cage-occupancy of methane, as presented in Supplementary Figure 16d.

Polycrystalline methane hydrates with randomly shaped grains

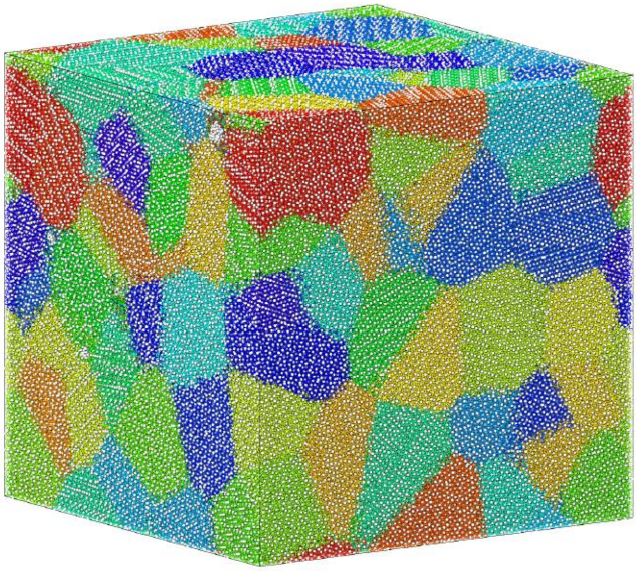

Supplementary Figure 12 | Representatively molecular model of polycrystalline methane hydrates with randomly shaped grains constructed by Voronoi method. The main manuscript mainly shows and discusses the results of mechanical instability of polycrystalline methane hydrates with uniformly geometrical grains. To exclude the origin of size-dependent mechanical instability of methane hydrates from specific uniformity of polycrystalline grains (grains are octahedral, 14-faced Archimedean solid with faces 8 {6} + 6 {4})), we construct another grain-textured polycrystalline methane hydrate which is composed of randomly geometrical grains. The average grain-size is around 15.0 nm, and grains are individually coloured for clarification. Methane molecules are highlighted by white-colouring particles. This figure shows a representatively 3-dimensional model of relaxed polycrystalline methane hydrates with randomly shaped grains. Few tiny bubbles are detected at the grain-junctions due to stress-induced dissociation, similar to that of polycrystal with uniform large-grains.

Stress-strain curves for polycrystalline methane hydrates with randomly shaped grains

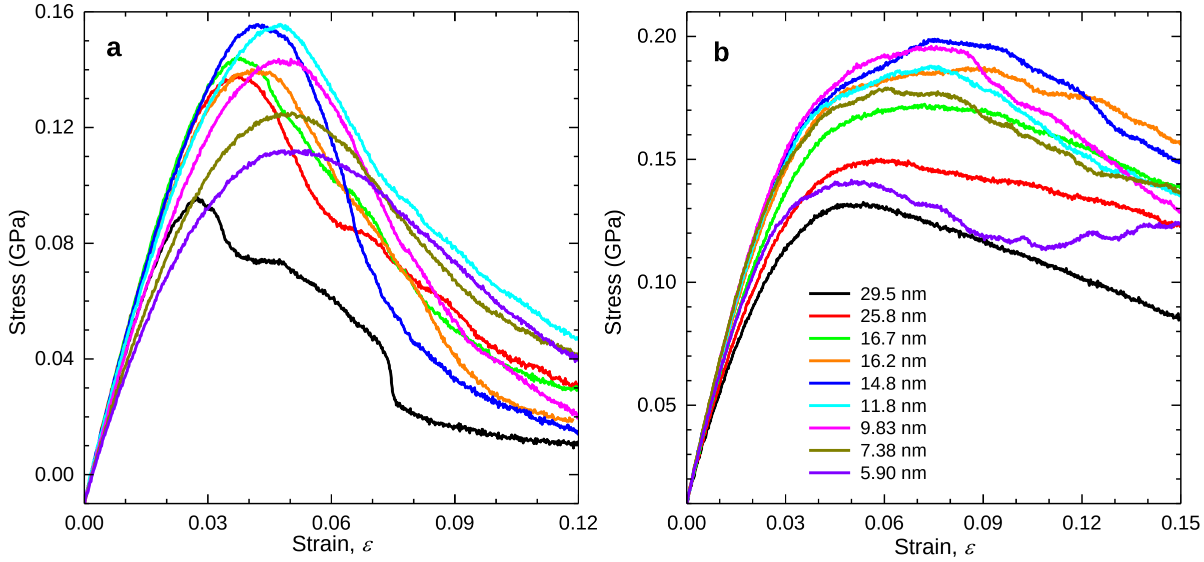

Supplementary Figure 13 | Predicted stress-strain curves for polycrystalline methane hydrates composed of randomly shaped grains with average grain-size from 5.90-29.5 nm subjected to (a) uniaxial tensile straining and **(b)** uniaxial compressive straining.

Mechanical properties of polycrystalline methane hydrates with randomly shaped grains

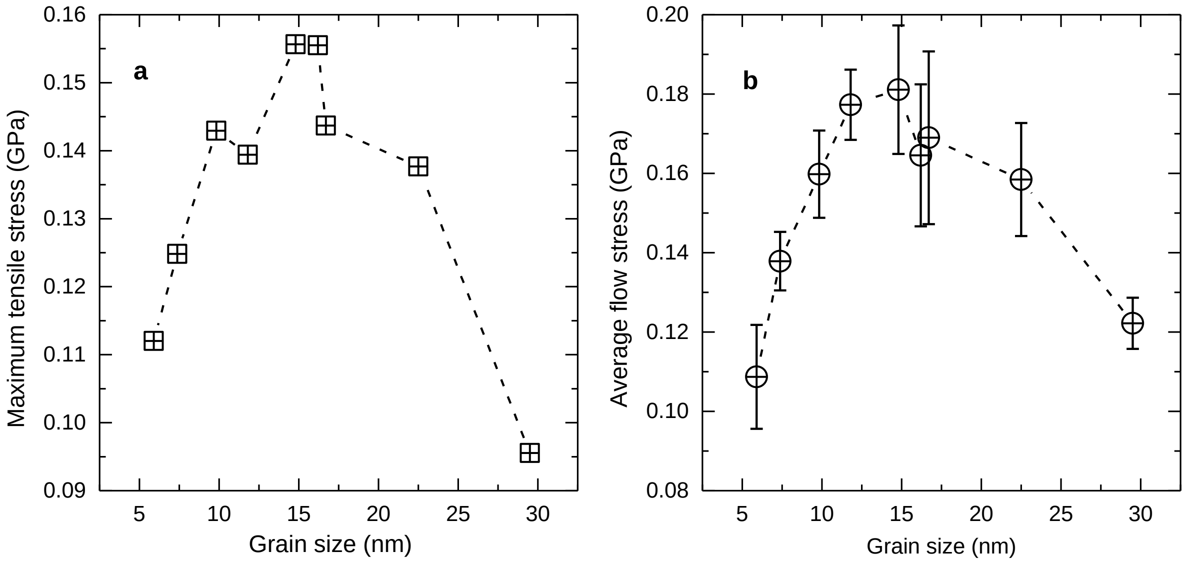

Supplementary Figure 14 | Mechanical properties of polycrystalline methane hydrates with randomly geometrical grains. (a) maximum tensile stress and **(b)** average compressive flow stress from engineering strain of 0.08-0.15 for polycrystalline methane hydrates with average grain-size from 5.90-29.5 nm. Overall, the values are slightly lower than those with uniform grains. This suggests that geometry of grains influences the mechanical instability (mechanical strength) of polycrystals due to distinct localised molecular structure of grain-boundary and grain-junction. Similarly, a transition of mechanical strength from strengthening to weakening takes place in polycrystals with randomly shaped grains. This again confirms the existence of both Hall-Petch and inverse Hall-Petch behavior in polycrystalline methane hydrates.

Disordered water for the hydrate polycrystals with uniform grains

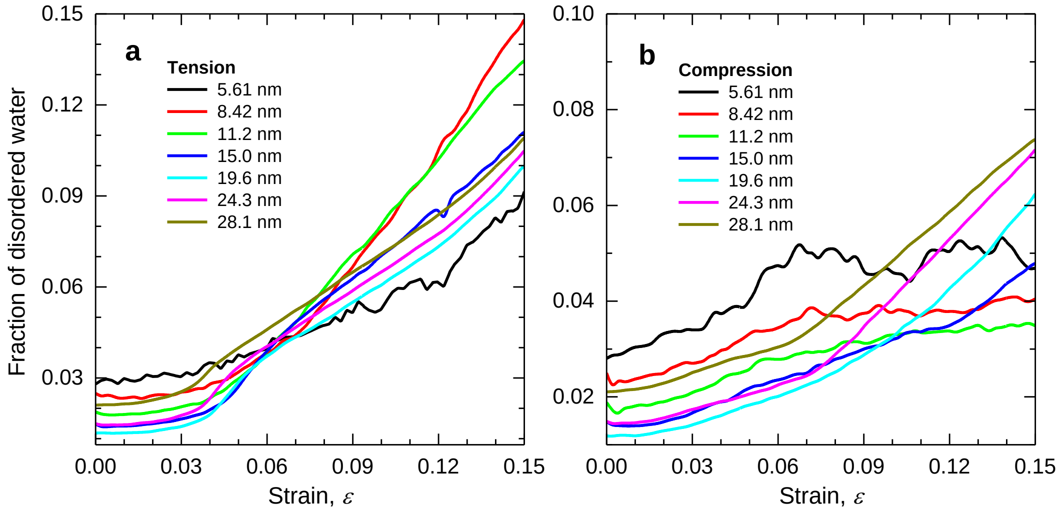

Supplementary Figure 15 | Fraction of disordered water in the simulated systems as a fucntion of (a) tensile strain and **(b)** compressive strain. The disordered water is defined by according to its cohesive energy. As shown in Fig.1c in the main manuscript, water possesses potential energy of less than -0.45eV prior to initiation of fracture. For conventional solid materials, the fraction of disordered atoms is proportional to the polycrystalline grain-size. However, it is observed from this figure that prior to uniaxial loading, the polycrystalline methane hydrates shows a crossover in the fraction of disordered water; it decreases with increasing of grain size to 19.6 nm, reaching a minimum value, whereas it increase as the grain becomes larger. This reflects the occurrence of hydrate dissociation in large-grained polycrystals, as shown in Manuscript Fig. 2. Under tension, initial elastic stretching almost does not lead to hydrate dissociation. Further elongation causes slight dissociation. When approaching the maximum tensile stress, deformation-induced dissociation speeds up to carry elastic-plastic deformation. Following the first rapid strain-softening, the fraction of disordered water is linearly proportional to the strain, representing a steady dissociation. Upon compression, when the grain is larger than 15.0 nm, an acceleration of compressive deformation-induced dissociation accompanied by grain-boundary sliding appears at a critical strain; however, as the grain-size falls below the value of 15.0 nm, the fraction of disordered water is considered to be constant posterior to a critical strain, indicating comparable larger-scale solid-state structural transformation occurring in the system, which strikingly differs from that subjected to tension. When the strain is over around 0.07, the global fraction of disordered water under tension exceeds those in compression. As discussed in main context, tensile strain-induced decohesion accompanied by coalescence of methane bubbles develops a markedly local depressurised environment which greatly promotes the decomposition of hydrate polycrystals.

Effect of cage occupancy on the mechanical responses

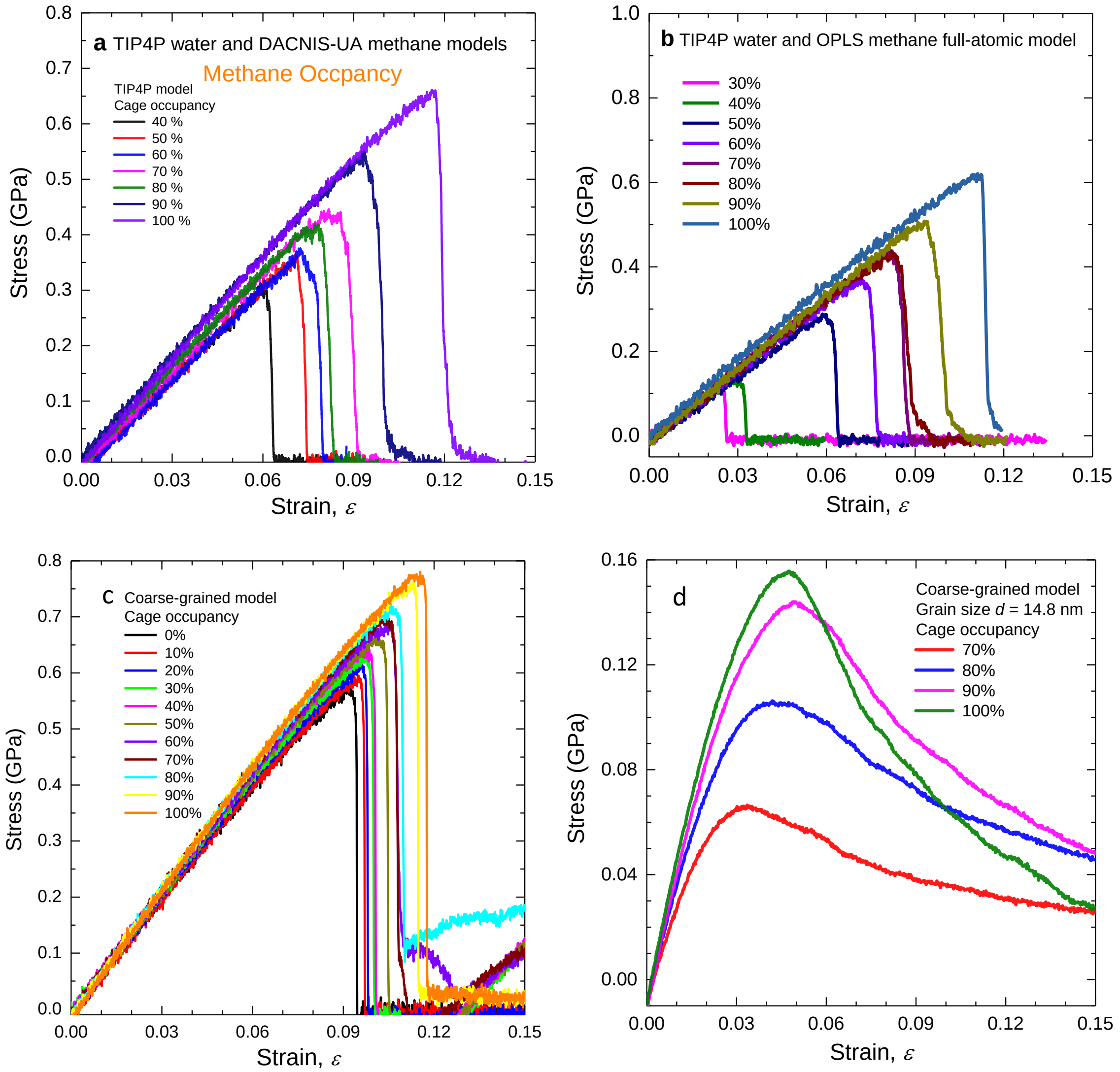

Supplementary Figure 16 | Effect of cage-occupancy on the mechanical instablity of methane hydrates under tensil. Predicted stress-strain relations of single-crystal by **(a)** TIP4P and DACNIS-UA models, **(b)** TIP4P and full-atomic OPLS models, and **(c)** coarse-grained model. **(d)** predicted stress-strain relations of polycrystalline methane hydrates by coarse-grained model. It can be summarized that methane molecules trapped inside the polyhedral water-cavities not only affects the molecular-level structures of grain boundary but also enhances the mechanical stability of polycrystaline hydrates.

Full-atomic polycrystalline methane hydrates

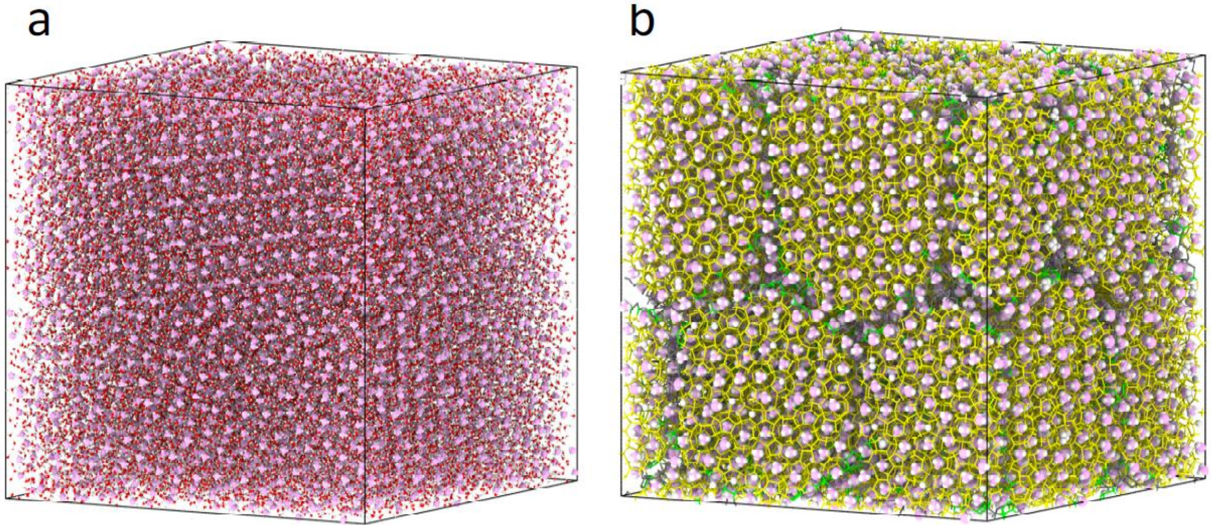

Supplementary Figure 17 | Full-atomic polycrystal model of methane hydrates with grain size of around 6 nm. (a) hydrogen: white, oxygen: red, carbon: lavender. (b) green: 5^{12} cages, yellow: $5^{12}6^{2}$ cages, grey: unidentified molecules, hydrogen of methane: white, carbon of methane: lavender. In the manuscript, we constructed both monocrystalline and polycrystalline methane hydrates with a coarse-grained model, and presented their mechanical properties. However, fully atomic models usually give more precise description of the molecular mechanisms at work during the deformation process. To justify the results obtained from the coarse-grained potential, we therefore prepared fully atomic models of both monocrystalline and polycrystalline methane hydrates for straining tests. For the case of monocrystalline methane hydrates, a 4×4×4 supercell, identical to coarse-grained model in the main text, was created to evaluate the mechanical response (Supplementary Figs.2-4). To further confirm the feasibility of the choice of coarse-grained model on the mechanical properties of polycrystalline methane hydrate, one full-atomic polycrystal model of methane hydrates with grain size of around 6 nm was prepared, as shown in Supplementary Figure 17. This fully atomic model of polycrystal consists of 43190 water molecules and 7290 methane molecules. The hydrate number can be calculated to be around 5.92. The construction procedure is identical to that of coarse-grained model of polycrystalline methane hydrates. To avoid unreasonable overlapping at the grain-boundaries, molecule (water and methane) which protrudes beyond the grain-boundaries was removed when a molecule pair with a nearest neighbor distance is less than 0.1 nm.

SPC/E water plus OPLS methane fully atomic models

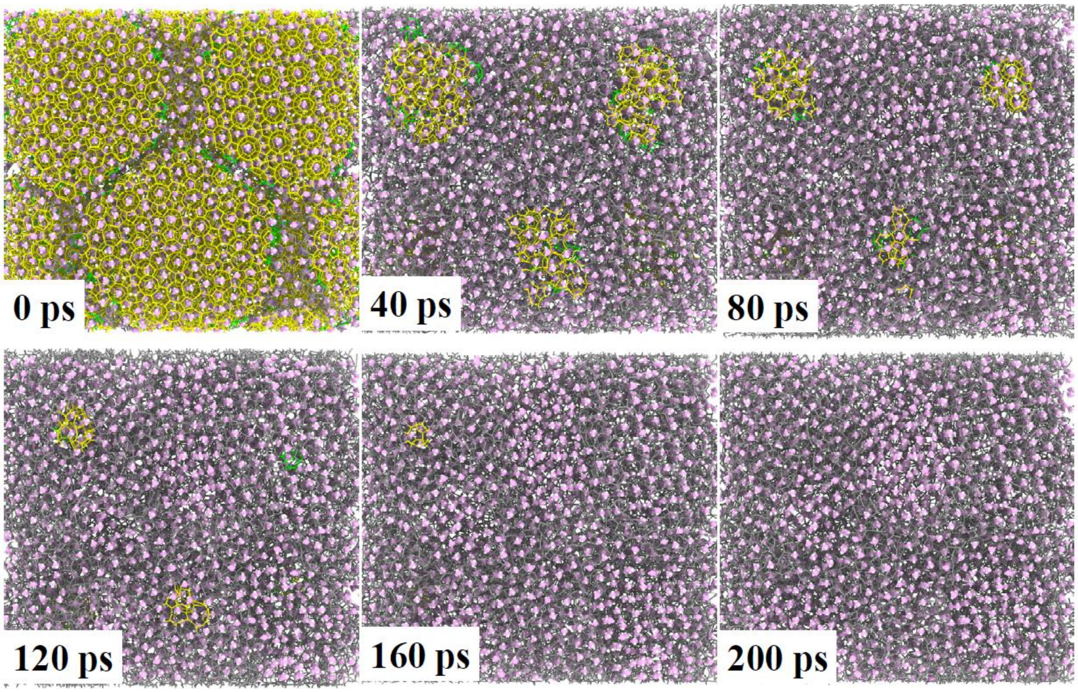

Supplementary Figure 18 | Structural developments of full-atomic polycrystal model of methane hydrate base on SPC/E water and OPLS methane models. It is found that both SPC/E and TIP4P water plus OPLS methane fully atomic models are not able to stabilise the polycrystal in the equilibrium simulation.

TIP4P/2005 water plus OPLS methane fully atomic models

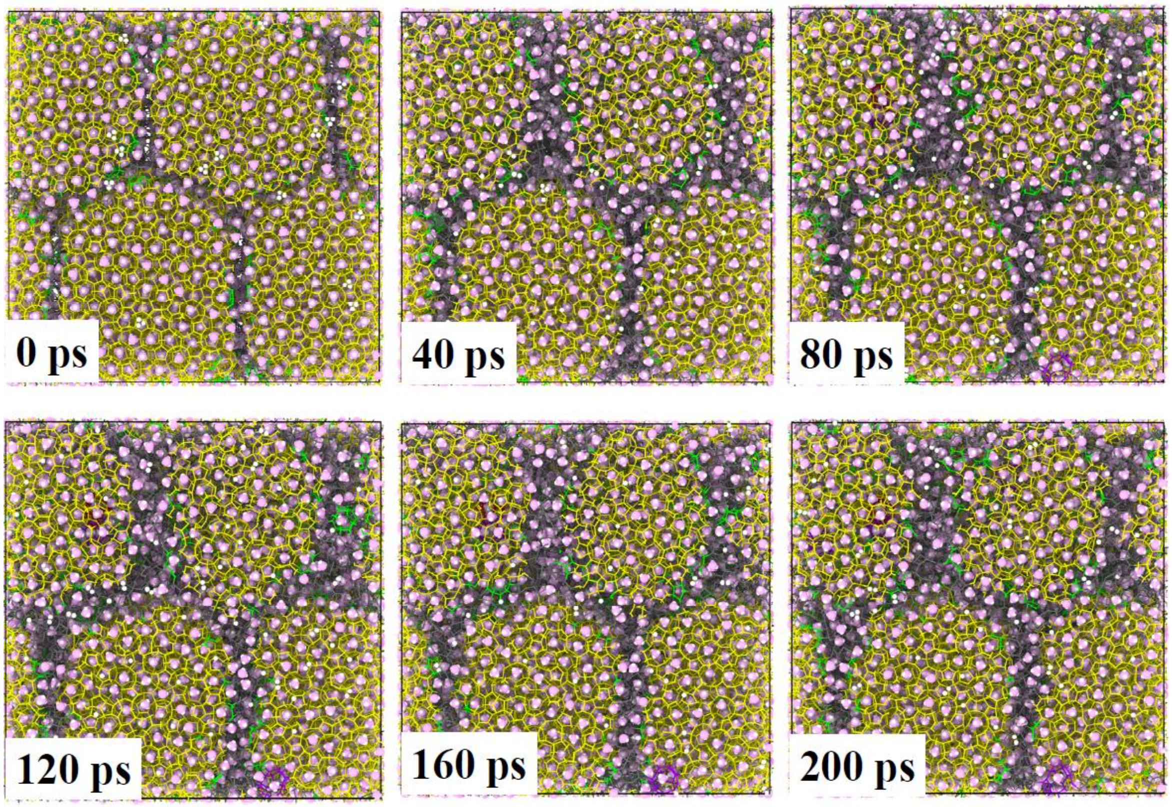

Supplementary Figure 19 | Structural developments of full-atomic polycrystal model of methane hydrate base on TIP4P/2005 water and OPLS methane models. Both the TIP4P/2005 and TIP4P/ICE water plus OPLS methane full-atomic models stabilise the polycrystal in the equilibrium simulation.

Mechanical instability of fully atomic polycrystalline methane hydrates with grain size of around 6 nm

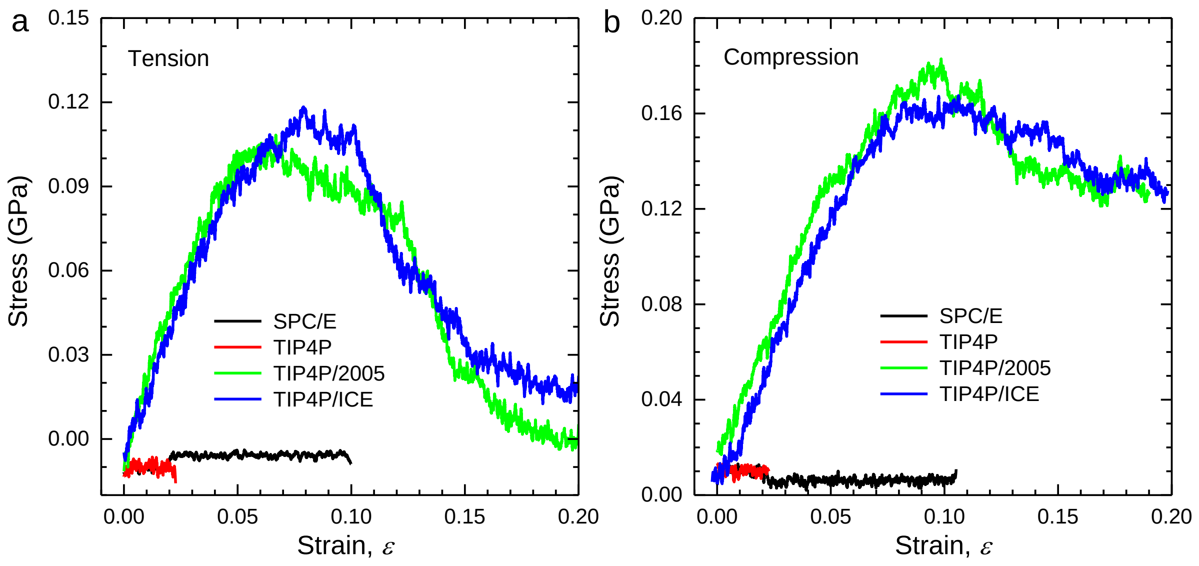

Supplementary Figure 20 | Mechanical responses of full-atomic polycrystalline methane hydrates predicted by four different fully atomic water and OPLS methane models subjected to (a) tension and (b) compression. To further confirm the feasibility of the choice of coarse-grained model on the mechanical properties of polycrystalline methane hydrates, we therefore prepared one full-atomic polycrystal model of methane hydrates with grain size of around 6 nm, as shown in Supplementary Figure 17. The resulting mechanical responses are shown in Supplementary Figure 20. Excluding the cases of SPC/E and TIP4P (see Supplementary Figure 18), the features of mechanical behaviours of polycrystalline *s*I methane hydrates by both TIP4P/2005 and TIP4P/ICE water plus OPLS full-atom models are similar to that by the monatomic model (See Fig.3a-b of manuscript). This again justifies the application of monatomic models for describing the mechanical behaviours of methane hydrates (Supplementary Figs.2-4).

Standard velocity-Verlet VS r-RESPA multi-timescale integrator

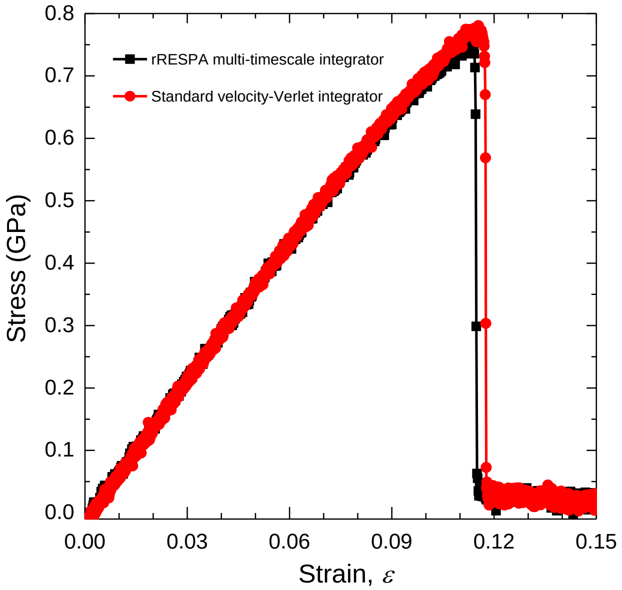

Supplementary Figure 21 | Comparison of stress-strain relations of methane hydrate based on r-RESPA and velocity-Verlet integrator. In our study, the reversible reference system propagator algorithm (r-RESPA) multi-timescale integrator to integrate the equations of motion was adopted to speed-up the simulations. To confirm the r-RESPA time-step set-up, another simulation was performed by using standard velocity-Verlet integrator to integrate the equations of motion during the deformation process. No difference in the deformational response clearly confirms the applicability of r-RESPA multi-timescale integrator in this work. The r-RESPA algorithm is theoretically Hamiltonian conservative. Furthermore, Nosé-Hoover thermostat and barostat employed to control the pressure and temperature in our study are able to conserve the Hamiltonian during the simulations.

Deformation control VS stress control technique

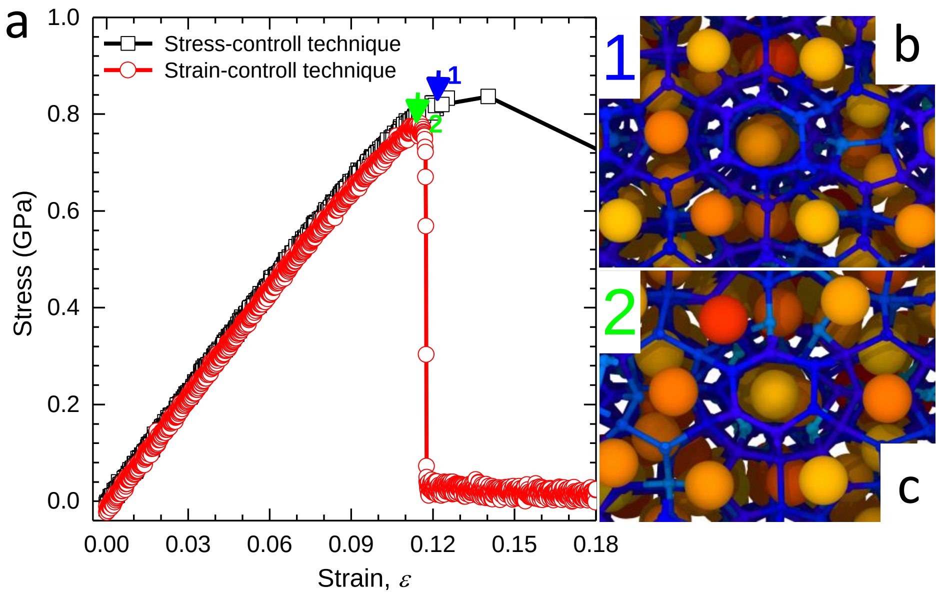

Supplementary Figure 22 | Deformation control and stress control technique on the mechanical responses of single crystal methane hydrate. (a) a comparison of stress-strain curves of single crystal methane hydrate obtained by strain-control and stress-control techniques. Snapshots of local molecular cages just before fracture by **(b)** strain-control and **(c)** stress-control methods.

Stress calculation by using force-across-a-plane / area of that plane

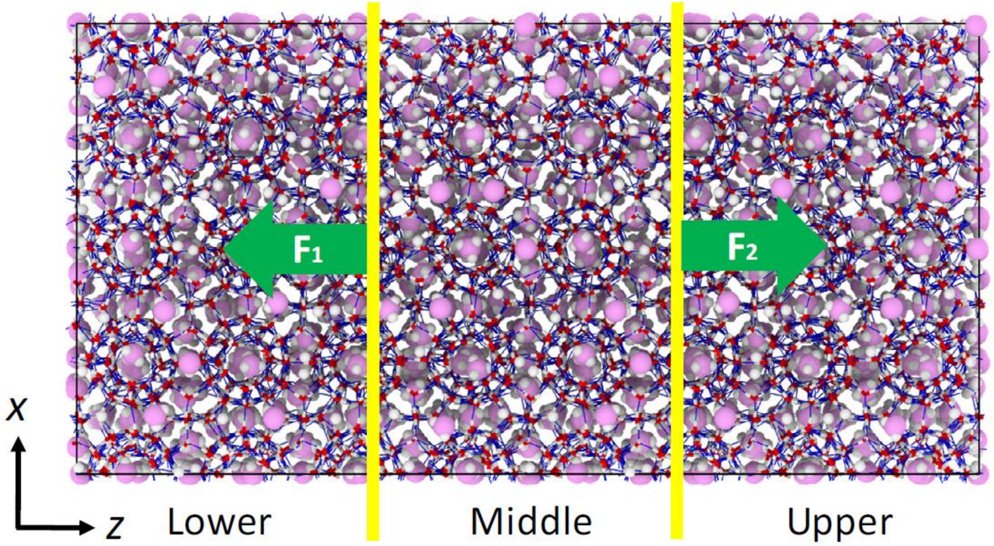

Supplementary Figure 23 | A 4×4×4 supercell model of monocrystalline methane hydrate for calculating stress by using force-across-a-plane / area of that plane. Blue line represents the hydrogen-bonding between water molecules. To confirm the method for calculating the mechanical stress in our study, we did another stress calculation by using force-across-a-plane / area of that plane during a uniaxial deformation. The principle can be concluded as follows. First, three sliced regions on the testing specimen (4×4×4 supercell) were made, where regions are perpendicular to the *z*-axis. The molecules falling in these three regions were defined as Lower, Middle, and Upper groups. It is noted that the thickness of these regions is larger than twice of the cut-off radius (12Å) to eliminate the boundary effect. Secondly, we defined a computation that calculates force interaction (F_1) between Middle and Lower groups of molecules, and (F_2) between Middle and Upper groups of molecules. The interaction forces F_1 and F_2 include the force on the computed group molecules due to Coulombic and vdW interactions with methane and water in the specified group. The final stress value can be calculated as $\sigma_z = (F_1 - F_2) / L_x / L_y$. L_x and L_y are the edge length of the simulated box along *x* and y dimensions, respectively. It is noted that $F_1/L_x/L_y + F_2/L_x/L_y = 0$, but $|(F_1 - F_2) / L_x/L_y| > 0$. The resulting stress-strain curves were shown in Supplementary Figure 24.

Stress calculated from force / area VS summing up viral stress

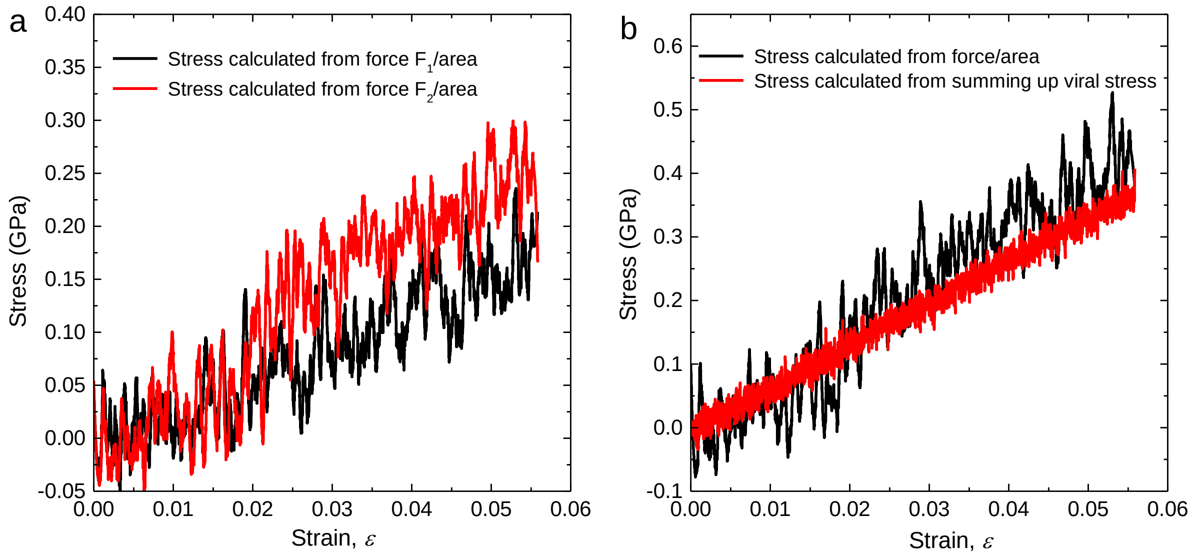

Supplementary Figure 24 | Stress-strain relationships of methane hydrates under the tensile condition. **(a)** stress calculated from force F_1/area and F_2/area, **(b)** stress calculated from $|(F_1 - F_2)/L_x/L_y|$ and summing up virial stress in the systems. It can be found that the interaction forces F_1 and F_2 are equal due to symmetrical structures, and applicability of virial stress based on the virial theorem of Clausius for calculating the true mechanical stress is reasonable.

Supplementary Note 1: Single-crystal Ice-I$_h$ VS single-crystal methane hydrate

Methane hydrate is ice-like in many respects. Simulations of uniaxial tension were also carried out for pure water ice by using the coarse-grained mW model [1], as adopted for methane hydrate in this study. Ice in the form of hexagonal structure (Ice-I$_h$, space group P63/mmc) was chosen for comparison with *s*I methane hydrate. Prior to mechanical loadings, the Ice-I$_h$ lattice was reconstructed into an orthorhombic structure with eight oxygen atoms per unit cell. Supplementary Figure 1 illustrates the molecular structure of single-crystal Ice-I$_h$ and the resulting stress-strain behavior in both single-crystal Ice-I$_h$ and methane hydrate. The data shows that both materials have only one elastic deformation behavior. This shows an abrupt drop in tensile stress, insensitive to the three orthogonal directions (Fig. 1a of the main manuscript), meaning a brittle failure pattern of both materials. The Ice-I$_h$ yields a slightly higher ultimate tension stress along the z direction, the <0 0 0 1> direction, than along the other two directions, in contrast to that of methane hydrate. However, the two critical strains for the three orthogonal directions are almost identical. Methane hydrate possesses both larger ultimate tensile stress and critical strain than that of Ice-I$_h$, whereas Ice-I$_h$ behaves slightly stiffer than methane hydrate.

Supplementary Note 2: *ab-initio* molecular dynamic (AIMD) simulations on the mechanical response of single crystal methane hydrate

The *ab-initio* molecular dynamic (AIMD) simulations were also performed by using Quickstep [2] of the CP2K program package to evauate the mechanical behaviours of methane hydrate. In the simulation, a Born-Oppenheimer molecular dynamic (BOMD) algorithm was empolyed. The Gaussian and plane waves (GPW) were used to describe molecules of methane hydrate [3]. In particular, the Goedecker-Teter-Hutter (GTH) pseudopotentials [4, 5] were used to describe atomic cores and the Becke-Lee-Yang-Parr (BLYP) exchange-correlation functional [6, 7], a double-zeta valence polarized (DZVP) basis set, was used [8]. We chose an energy cut-off of 400 Ry for the additional plane wave basis sets. A time step of 0.5 fs was used in the AIMD simulation. The electronic energy was converged to 10^{-6} hartree with the orbital transformation method [9]. To describe the van der Waals (vdW) interactions, an empirical dispersion correction of Grimme's type was applied [10]. This set up was proven to yield accurate energies and dynamics for hydrates[11]. A thermostat and barostat (N*p*T) algorithm was applied to control the temperature at 283.15 K and the pressure at 10 MPa. Since AIMD is much more expensive than classical MD, a smaller system consisting of a 2×1×1 supercell (356 atoms) of methane hydrate was therefore simulated. Comparing with classic MD simulations, a high strain rate of 2×10^{9} /s was applied. The total simulation time to construct the stress-strain curve was 320 ps.

Supplementary Figure 5 shows the stress-strain curve of single crystal methane hydrate from AIMD simulation together with one snapshot captured at strain of 0.2. It is observed that the characterstic of the strain-stress curve is similar with those obtain from classical MD. The Young's modulus was calculated to be around 9.9 GPa, consistent with those of classical MD calculations. The ultimate stress and the critical strain

obtained from AIMD simulation with high strain rate were comparable to those of classic MD simulation. Movie S4 recorded the entire deformation process.

Supplementary Note 3: Dynamical properties of a fractured monocrystalline methane hydrate by the coarse grained model

Supplementary Figure 4b plots the variation in hydrogen-bonds per unit cell during the straining. We have defined existence of hydrogen-bond in the fully atomistic model with a donor-acceptor distance and an angle cutoff set to 3.0 Å and 120º. For the coarse-grained model the distance of donor-acceptor (i.e. monotonic water to water particle) was set to 3.0 Å, without limitation in the angle cutoff. All simulations display the same tendency. A reduction of hydrogen-bonds of around 4-6%, depending on the models of water, results in the initiation of hydrate fracture, which compares that a melting of solid water ice breaks 15% of hydrogen-bonds [12]. Prior to the onset of fracture, elastic stretching misshapes both large and small hydrate cages leading to reduction of hydrogen-bonds. The catastrophic fracture instantly releases the straining energy and leads to that misshapen cage recovery to their original geometry. This partial recovery enhances the reformation of hydrogen-bonds, as signified by a climb in the curves.

Mean square displacement (MSD) is a useful tool to describe the dynamical properties of a system. To confirm that water molecules in the interstice of fractured methane hydrate is in liquid-like state, we further did two additional MD simulations to extract the time-dependent MSDs of water and methane molecules in the fractured methane hydrate system. In the simulation setup, two groups (non-destructed and decomposed methane hydrate) were defined as shown in the inset of Supplementary Figure 10. MD simulations of 100 ns were carried out under NVT ensemble with Nosé-Hoover thermostat. Supplementary Figure 10 presents a comparison of MSDs of water and methane molecules in the interstice of fractured hydrate and in the non-destructive hydrate. Obviously, water and methane molecules in the interstice of fractured hydrate show larger MSDs values than those in the non-destructive hydrate. This predicted larger MSD of methane molecules in the interstice of fractured hydrate is indicative of that these methane molecules are in gaseous state. Also, very small MSD values of water and methane molecules in the non-destructive hydrate indicate that these molecules are in solid state. Accordingly, intermediate MSD value of water molecules in the interstice of fractured hydrate implies that these water molecules are in liquid-like state.

Supplementary Note 4: Effect of cage occupancy on the mechanical response of methane hydrates

The presence of guest molecules encapsulated in water-cages of gas hydrates plays a central role on their stability; vdW interactions between water and guest molecules prevent hydrogen-bonded strain and breakage. Experimental studies determined that the cage-occupancy of both 12-hedral and 14-hedral cages in *s*I methane hydrate is very high, and the hydrate number varies from around 5.65-7.4 [13-15]. We performed several simulations to evaluate the effect of cage-occupancy on the mechanical instability of both single-crystal and polycrystals subjected to uniaxial straining. Molecular models of incompletely occupied methane hydrates were achieved by random deletion of methane in both large and small cages without control of hydrate number. Prior to mechanical loadings, simulations with a sufficient equilibrium time under N*p*T ensemble were conducted. Supplementary Figure 16 shows their corresponding stress-strain behaviors. In the case of single crystal described by TIP4P model plus DACNIS-UA [16] or full-atomic OPLS methane model [17], the absence of stress-strain relations of methane hydrates with cage-occupancy of 0-30% or 0-20% in Supplementary Figure 16a or b is due to their destabilization in the relaxing process. Mechanical responses of single crystal predicted by fully atomistic model (TIP4P) with full/united methane model and coarse-grained model in Supplementary Figure 16a-c reveal that low cage occupancy remarkably weakens the materials, suggesting significance of guest molecules on the materials stability of gas hydrates from mechanical point of view. Both natural and synthetic gas hydrates are unavoidably polycrystalline. Similarly, the mechanical stability of polycrystalline methane hydrates is highly sensitive to the cage-occupancy. As shown in Supplementary Figure 16d, the ultimate strength of polycrystalline methane hydrates with grain size of 14.8nm and a cage-occupancy of 70% is over one fold lower than that of polycrystal with a cage-occupancy of 100%. This illustrates high-energetic grain-boundary greatly constraints their cage-occupancy. Higher cage-occupancy in both natural and synthetic polycrystalline methane hydrates measured by experiments may come from *P*-*T* condition, large size of grain, porosity and impurity of samples.

Supplementary Note 5: MD simulations with fully atomic models

In order to examine the influence of force fields on the calculated mechanical properties, four differently full-atomic water (SPC/E, TIP4P, TIP4P/2005, TIP4P/ICE) models plus a DACNIS united-atom (DACNIS-UA) methane model [16] or a full-atomic OPLS (Optimized Potentials for Liquid Simulations) methane model [17] were employed to describe interactions of the monocrystal and polycrystal systems. The cutoff of short-range van der Waals (vdW) interactions was set to be 12.0 Å. The particle-particle-particle-mesh (PPPM) algorithm was applied to take into account the long-range Coulombic electrostatics interactions in the methane hydrate systems. The Shake algorithm was used to fix the bond and angle between the atoms in water molecules (all water molecules were taken as rigid) with a relative shake tolerance of 1.0×10^{-4}. Similar to the case of coarse-

grained methane hydrate, the energy minimization of the fully atomic methane hydrates system was achieved by the Polak-Ribiere conjugate gradient method [3] with an energy tolerance of 1.0×10^{-4} eV and a force tolerance of 1.0×10^{-6} eV/Å. In MD simulations, Newton's equations of motion were numerically integrated for all atoms by means of the standard velocity-Verlet algorithm. The integration time step was 1 fs. The MD relaxation was carried out with a simulation time of 200 picoseconds under cold (T = 283.15 K) and pressurised (p = 10 MPa) conditions in the NpT ensemble. The pressure and temperature were controlled by using the Nosé-Hoover barostat and thermostat with damping time constant τ_T = 2 ps and τ_p = 5 ps. In the equilibrium simulation, it is found that both SPC/E and TIP4P water plus OPLS methane fully atomic models are not able to stabilise the polycrystal, as shown in Supplementary Figure 18. However, both the TIP4P/2005 and TIP4P/ICE water plus OPLS methane full-atomic models stabilise the polycrystal, as shown in Supplementary Figure 19. Additionally, both tension and compression simulation tests on this polycrystal were further performed to evaluate its mechanical response. The uniaxial loading was simulated by the deformation control technique. The deformation operation was imposed by adjusting the simulation cell size in z direction and remapping all atoms to the new box by a simple rescaling of all coordinates. The stepwise loading step and tensile strain are 100 timesteps and 0.00001/ps, respectively, identical to the case of coarse-grained structures. This deformation simulation corresponds to a modified NpT ensemble with anisotropic barostat and thermostat of Nosé-Hoover, namely *NVT* in the loading direction, and NpT in the lateral directions, allowing the simulation box to experience a Poisson expansion/contraction in the transverse directions.

Supplementary Note 6: Deformation control VS stress control technique on the mechanical responses of single crystal methane hydrate

A reasonable technique utilized to deform solid structures is the key to investigate mechanical properties of materials. The deformation control technique combined with $NZ_z p_x p_y T$ ensemble is able to provide a complete picture of mechanical characteristics of solid materials, although this technique may be slightly different from physical, experimental reality of applied tension or compression. To confirm this technique applied on elongation of methane hydrates, we employed stress-control or force-control technique to deform the materials. Anisotropic barostat and thermostat of Nosé-Hoover were used in MD simulations to maintain pressure in lateral directions independently and temperature, while pressure along the stretching direction gradually decreases to achieve a tension stress. It is noted that the total MD simulation steps with pressure decrease from 0.01 to -0.80 GPa are identical to that with strain increase from 0 to 0.12. That is to say, the average strain-rates between the two techniques are equivalent. The stress-strain responses of methane hydrates attained by strain-control and stress-control techniques are shown in Supplementary Figure 22. By comparison, there is no difference of mechanical responses in the elastic region between these two techniques. A large interval between square-dots in the plastic region for the case of stress-control technique is attributed to that applied

stress exceeding the critical fracture stress of methane hydrate leads to the separation of frustrated hydrate in a very short simulation time. Furthermore, no difference in the molecular structures of deformed water-cages prior to fracture between these two loadings is detected. This finding is in accordance with the understanding in mechanics. In tensile testing of materials, as long as the strain rate is the same, no difference between the two techniques should be expected before the maximum load is reached. The deformation (strain)-controlled technique is overwhelmingly used because load-controlled technique has challenges to follow the unloading curve and will lead to premature fracture and separation of material. In finite element simulation with commercial software ABAQUS, a special algorithm/method called RIKS has to be applied to deal with unloading if a stress-controlled technique has to be used.

Supplementary References

[1] Molinero, V., Moore, E. B. Water Modeled As an Intermediate Element between Carbon and Silicon. *J. Phys. Chem. B* **113**, 4008-4016(2009).

[2] VandeVondele, J., Krack, M., Mohamed, F., Parrinello, M., Chassaing, T., Hutter, J. QUICKSTEP: Fast and accurate density functional calculations using a mixed Gaussian and plane waves approach. *Comput. Phys. Commun.* **167**, 103-128(2005).

[3] Lippert, G., Hutter, J., Parrinello, M. A hybrid Gaussian and plane wave density functional scheme. *Mol. Phys*.**92**, 477-488(1997).

[4] Goedecker, S., Teter, M., Hutter, J. Separable dual-space Gaussian pseudopotentials. *Phys. Rev. B* **54**, 1703-1710(1996).

[5] Hartwigsen, C., Gœdecker, S., Hutter, J. Relativistic separable dual-space Gaussian pseudopotentials from H to Rn. *Phys. Rev. B* **58**, 3641–3662 (1998).

[6] Becke, A. D. Density-functional exchange-energy approximation with correct asymptotic-behavior. *Phys. Rev. A* **38**, 3098-3100(1988).

[7] Lee, C., Yang, W., Parr, R. G. Development of the Colle-Salvetti correlation-energy formula into a functional of the electron density. *Phys. Rev. B* **37**, 785-789(1988).

[8] VandeVondele, J., Hutter, J. Gaussian basis sets for accurate calculations on molecular systems in gas and condensed phases. *J. Chem. Phys*. **127**, 114105(2007).

[9] VandeVondele, J., Hutter, J. An efficient orbital transformation method for electronic structure calculations. *J. Chem. Phys.* **118**, 4365(2003).

[10] Grimme, S. Semiempirical GGA-type density functional constructed with a long-range dispersion correction. *J. Comput. Chem.* **27**, 1787-1799(2006).

[11] Zhao, W., Wang, L., Bai, J., Francisco, J. S., Zeng, X. C. Spontaneous formation of one-dimensional hydrogen gas hydrate in carbon nanotubes. *J. Am. Chem. Soc.* **136**, 10661-8(2014).

[12] Pauling, L. The Nature of the Chemical Bond, Cornell University. Press, Ithaca, New York (1945).

[13] Ripmeester, J. A., Ratcliffe, C. I. Low-temperature cross-polarization/magic angle spinning carbon-13 NMR of solid methane hydrates: structure, cage occupancy, and hydration numbe. *J. Phys. Chem.* **92**, 337-339(1988).

[14] De Roo, J. L., Peters, G. L., Lichtenthaler, R. N., Diepen, G. A. M. Occurrence of Methane Hydrate in Saturated and Unsaturated Solutions of Sodium Chloride and Water in Dependence of Temperature and Pressure. *AIChE J.* **29**, 651-657(1983).

[15] Glew, D. N. Aqueous Solubility and the Gas-Hydrates. The Methane-Water System1. *J. Phys. Chem*. **66**, 605-609(1962).

[16] Martin, M. G., Thompson, A. P., Nenoff, T. M. Effect of pressure, membrane thickness, and placement of control volumes on the flux of methane through thin silicalite membranes: A dual control volume grand canonical molecular dynamics study. *J. Chem. Phys.* **114**, 7174-7181(2001).

[17] George K., Erin M. D., Tooru M., William L. J. Free Energies of Hydration and Pure Liquid Properties of Hydrocarbons from the OPLS All-Atom Model. *J. Phys. Chem.* **98**, 13077-13082(1994).

[18] Navon I. M., Legler D. M. Conjugate-Gradient Methods for Large-Scale Minimization in Meteorology. *Mon. Wea. Rev.***115**, 1479-1502(1987).
